# *In vivo* monitoring of tissue regeneration using a ratiometric lysosomal AIE probe†

**DOI:** 10.1039/c9sc06226b

**Published:** 2020-02-11

**Authors:** Xiujuan Shi, Neng Yan, Guangle Niu, Simon H. P. Sung, Zhiyang Liu, Junkai Liu, Ryan T. K. Kwok, Jacky W. Y. Lam, Wen-Xiong Wang, Herman H.-Y. Sung, Ian D. Williams, Ben Zhong Tang

**Affiliations:** HKUST-Shenzhen Research Institute No. 9 Yuexing 1st RD, South Area, Hi-tech Park, Nanshan Shenzhen 518057 China tangbenz@ust.hk wx.wang@cityu.edu.hk chryan@ust.hk; Department of Chemical and Biological Engineering, Department of Chemistry, Hong Kong Branch of Chinese National Engineering Research Center for Tissue Restoration and Reconstruction, Institute for Advanced Study, The Hong Kong University of Science and Technology Clear Water Bay Kowloon Hong Kong China; Department of Ocean Science, The Hong Kong University of Science and Technology Clear Water Bay Kowloon Hong Kong China; School of Energy and Environment, State Key Laboratory of Marine Pollution, City University of Hong Kong Kowloon Hong Kong China; Ming Wai Lau Centre for Reparative Medicine, Karolinska Institute Hong Kong China; Centre for Aggregation-Induced Emission, SCUT-HKUST Joint Research Laboratory, State Key Laboratory of Luminescent Materials and Devices, South China University of Technology Guangzhou 510640 China

## Abstract

Tissue regeneration is a crucial self-renewal capability involving many complex biological processes. Although transgenic techniques and fluorescence immunohistochemical staining have promoted our understanding of tissue regeneration, simultaneous quantification and visualization of tissue regeneration processes is not easy to achieve. Herein, we developed a simple and quantitative method for the real-time and non-invasive observation of the process of tissue regeneration. The synthesized ratiometric aggregation-induced-emission (AIE) probe exhibits high selectivity and reversibility for pH responses, good ability to map lysosomal pH both *in vitro* and *in vivo*, good biocompatibility and excellent photostability. The caudal fin regeneration of a fish model (medaka larvae) was monitored by tracking the lysosomal pH change. It was found that the mean lysosomal pH is reduced during 24–48 hpa to promote the autophagic activity for cell debris degradation. Our research can quantify the changes in mean lysosomal pH and also exhibit its distribution during the caudal fin regeneration. We believe that the AIE-active lysosomal pH probe can also be potentially used for long-term tracking of various lysosome-involved biological processes, such as tracking the stress responses of tissue, tracking the inflammatory responses, and so on.

## Abstract 2

An AIE-active ratiometric probe for the first time achieved the long-term quantification of lysosomal pH during the medaka larva's caudal fin regeneration.

## Graphical abstract

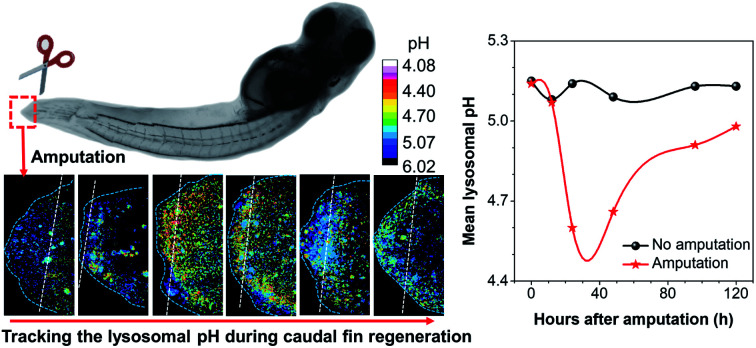

## Introduction

Tissue regeneration is the process of self-renewal and self-restoration to regrow damaged or lost body parts after injury.^1^ Although some human tissues including skin, fingertips, endometrium and liver can regenerate, this regenerating ability pales in comparison to that of some vertebrates like salamanders, zebrafish, Xenopus and so on.^2,3^ To facilitate the development of regenerative medicine, many attempts have been made to study the biological processes involved in tissue regeneration by using vertebrate genetic models.^4–7^ Among them, zebrafish and medaka are the widely used ones. Their central nervous system, fin, heart and kidney can regenerate; in particular, their caudal fin can regenerate with unlimited potential after amputation.^1,8–10^ They are good genetic model systems,^11–13^ and are easy to raise in the laboratory. Besides, they have a transparent body at the early stage of life, making it easy to carry out the *in vivo* imaging of tissues without the need to sacrifice the experimental subjects.^14–17^

In order to study the biological processes during tissue regeneration, the widely used method is fluorescence imaging, which has the advantages of high contrast, high sensitivity, dynamic imaging, low cost, ease of use and safety.^18–20^ One popular method of fluorescence imaging is through the application of transgenic fishes carrying a fluorescent-protein-labelled protein.^6,12^ Although the transgenic technique of fluorescent protein fusion provides the possibility to study protein dynamics in living cells, the technique is complicated, laborious, expensive and susceptible to photobleaching. And the large size of the fluorescent protein may affect the function of the fused protein.^21–23^ Another popular method of fluorescence imaging is fluorescence immunohistochemical staining,^7,21,22^ but it requires cell fixation and permeabilization, which can possibly lead to defects, including protein extraction or relocalization, epitope blockage and inability to perform real-time imaging. Thus, information obtained about certain biological processes may be incorrect or missing.^21,22^ Therefore, a simple method is highly demanded for real-time visualization of the process of tissue regeneration, which will help further verify the generally accepted but not wholly revealed biological changes.

Lysosomes are a well-known ‘garbage disposal system’ of cells because they are responsible for intracellular degradation of materials (damaged organelles, used proteins, DNA, phospholipids and so on), and also responsible for recycling metabolites and ions to maintain the homeostasis of cells. Therefore, they are involved in many important cellular processes and disease pathogenesis.^24–28^ Among them, one of the most important functions of lysosomes is to participate in autophagy, which is a lysosome-mediated self-degradation process of eukaryotic cells, and is indispensable to tissue regeneration.^6,29^ Lysosomal pH is an important parameter to determine the activity of lysosomal hydrolases and the lysosomal functions. To ensure that lysosomal hydrolases operate optimally, it is essential to maintain the lysosomal pH in an acidic range of pH 4–5.^30–32^ In some cases, if the lysosomal pH is not considered, it is possible to draw erroneous conclusions about the biological process investigated only by using the transgenic technique of fluorescent protein fusion or fluorescence immunohistochemical staining.^33^ For example, an increase in the expression of a fluorescent-protein-labelled LC3-II protein in autophagosomal membranes does not necessarily indicate an increase in autophagy flux, as it may be caused by the alkalization of lysosomes that hinders autophagosome degradation.^34^ Therefore, real-time tracking of lysosomal pH can help understand the events that happened during tissue regeneration to a more comprehensive extent.

Small-molecule fluorescent probes have become powerful tools for biological detections due to the advantages of easy synthesis, wide varieties, simple operation, high selectivity, fast recognition, and so on.^23,35,36^ Among the fluorescent probes, intensity-based fluorescent probes are not suitable for quantitative analysis because they are affected by many factors, including probe concentration variation and uneven distribution, temperature, environmental polarity, and excitation light fluctuation.^37–41^ In contrast, ratiometric probes can overcome the systematic errors and are more accurate for quantification.^42–49^ Conventional aggregation-caused quenching (ACQ) probes have the problem of fluorescence quenching at high concentration or in the aggregated state due to the strong intermolecular π–π interactions. Therefore, ACQ probes of low concentrations are suggested to be applied in biological applications.^50–52^ But this brings other problems, such as easy photobleaching and being not applicable for long-term tracking. Moreover, their small Stokes shift can result in strong self-absorption.^53,54^ In contrast to ACQ materials, the greater the quantity of AIE materials, the more intensely they illuminate. In recent decades, AIEgens have been proved to possess high photostability, large Stokes shift, excellent capability of long-term tracking and good biocompatibility.^55–67^ Although great attention has been paid to tissue regeneration and lysosome study,^4,5,27,28,68–70^ monitoring lysosomal pH during tissue regeneration *in vivo* has not been investigated yet.

In general, a good fluorescent probe for tracking lysosomal pH during tissue regeneration needs to meet the following requirements: (1) good photostability; (2) long-term tracking ability; (3) lysosome targeting; (4) high pH sensitivity; (5) quantitative measurement; (6) non-invasive *in vivo* imaging. Herein, a ratiometric lysosomal pH probe with AIE properties was developed, and the probe can fulfil all the requirements listed above. In terms of the molecular design, the α-cyanostilbene-based structure was used to endow the molecule with AIE properties. The piperazine group is used for lysosome targeting. The large change of intramolecular charge transfer during pyridine protonation enables us to ratiometrically quantify the lysosomal pH. The performance of the AIE probe in pH sensitivity, lysosome targeting, biocompatibility, photostability and lysosomal pH measurement was first examined *in vitro*. Then, its good performances in experiments *in vitro* encouraged us to further explore its performance in mapping lysosomal pH *in vivo*. To demonstrate our probe's capability of tracking tissue regeneration, the caudal fin of the medaka larva was amputated, and the process of regeneration was monitored by tracking the lysosomal pH change after amputation.

## Results and discussion

### The AIE properties of CSMPP

The cyanostylbene derivative, (*Z*)-3-(4-(4-methylpiperazin-1-yl)phenyl)-2-(4-(pyridin-4-yl)phenyl)acrylonitrile, abbreviated as CSMPP, was synthesized through two simple steps: Suzuki coupling reaction and Knoevenagel condensation reaction. The synthetic scheme, nuclear magnetic resonance (NMR) spectra and high-resolution mass spectrum of CSMPP are given in Scheme S1 and Fig. S1–S3 in the ESI.†

**Fig. 1:**
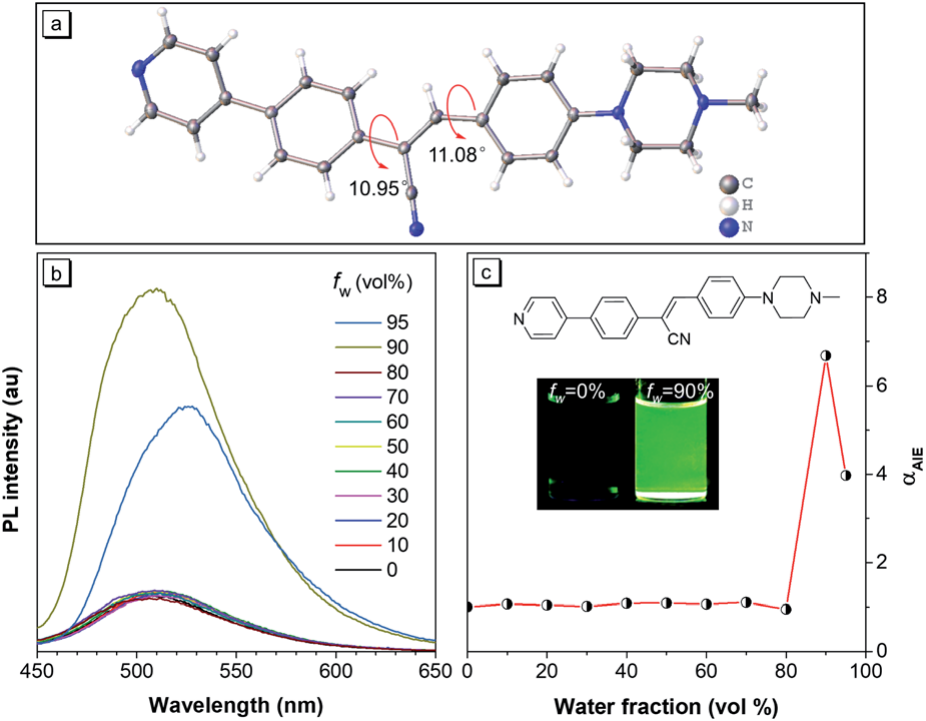
(a) The single crystal structure of CSMPP. (b) PL spectra of CSMPP in acetonitrile/water mixtures with different volume fractions of water (*f*_w_). (c) Plot of *α*_AIE_*versus* water fraction. *α* is *I*/*I*_0_. *I* and *I*_0_ represent the PL intensities at 509 nm in the acetonitrile/water mixture with a specific *f*_w_ and in pure acetonitrile, respectively. The concentration of CSMPP is 10 μM. Excitation wavelength is 365 nm. Insets in graph c are the fluorescence images of CSMPP solutions with *f*_w_ = 0% and *f*_w_ = 90% under a hand-held UV lamp with an excitation wavelength of 365 nm. The chemical structure of CSMPP is inserted in the graph c.

As shown in Fig. 1b, 10 μM CSMPP solution in acetonitrile exhibited a very weak emission centred around 509 nm with an absolute fluorescence quantum yield of 0.8%. The photoluminescence (PL) of CSMPP as shown in Fig. 1c exhibited no obvious change when the water fraction (*f*_w_) in the mixture of acetonitrile and water was below 80%, but sharply increased with further addition of water due to the formation of aggregates (Fig. S4†). The PL intensity of CSMPP at 90% *f*_w_ was about 7-fold that at 0% *f*_w_, indicating the aggregation-induced emission (AIE) properties. In addition, the absolute fluorescence quantum yield of CSMPP powder reached 25.4% with an emission peak at 525 nm. Apart from aggregation, the PL of CSMPP can also be triggered in a highly viscous liquid owing to the restriction of the intramolecular motion (RIM) of AIE molecules that promotes radiative decay. As shown in Fig. S5,† with the increase of glycerol fraction in the mixture of glycerol and ethylene glycol, the PL emission of CSMPP gradually increased. And the CSMPP solution containing 95% glycerol showed a quantum yield of 10.5%.

To further understand the AIE properties of the CSMPP molecule, its single crystal structure was analyzed (Fig. 1a). The molecule adopted a slightly twisted conformation with dihedral angles of 10.95° and 11.08°. Thus, the phenyl rings adjacent to the acrylonitrile group can rotate freely in solvent, which dissipated the energy of the excitons in a non-radiative way. And the RIM mechanism of AIE can be activated when the molecules are in the aggregated state or in a highly viscous solution, thus endowing the molecules with strong emission.

### Ratiometric pH measurements

**Fig. 2:**
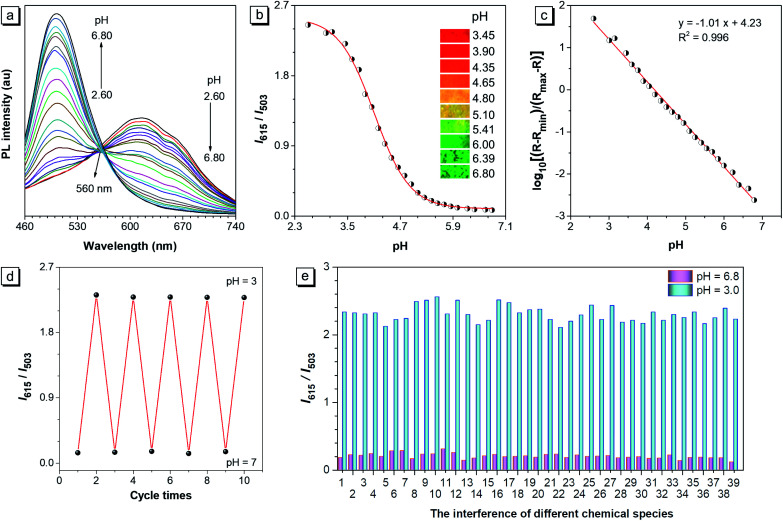
(a) Fluorescence spectra and (b) fluorescence intensity ratios (*I*_615_/*I*_503_) of CSMPP (10 μM) in buffers (containing 2% DMSO) in the pH range of 2.60–6.80. The inset fluorescence pictures are the solutions at different pH taken under a hand-held UV lamp with an excitation of 365 nm. (c) The calibration curve was fitted based on the relationship between the ratio (*I*_615_/*I*_503_), *R*_min_, *R*_max_ and the pH, whose equation is presented in the ESI.† (d) The pH reversibility of CSMPP in response to pH 7 and pH 3. The PL intensity ratio (*I*_615_/*I*_503_) is plotted as a function of cycle times. (e) The interference of different chemical species with the probe in the buffer of pH 6.8 and pH 3.0. Chemical species: (1) blank; (2) Ca^2+^; (3) Mg^2+^; (4) Cd^2+^; (5) Co^2+^; (6) Fe^2+^; (7) Fe^3+^; (8) Ni^2+^; (9) Cu^2+^; (10) Al^3+^; (11) Zn^2+^; (12) Ag^+^; (13) Mn^2+^; (14) Pb^2+^; (15) K^+^; (16) Na^+^; (17) I^−^; (18) HPO_4_^2−^; (19) H_2_PO_4_^−^; (20) Ac^−^; (21) NO_3_^−^; (22) CO_3_^2−^; (23) ClO^−^; (24) HS^−^; (25) SCN^−^; (26) S_2_O_3_^2−^; (27) H_2_O_2_; (28) tryptophan; (29) glutamine; (30) aspartic acid; (31) glycine; (32) leucine; (33) valine; (34) cysteine; (35) homocysteine; (36) glutathione; (37) arginine; (38) serine; (39) glucose. Condition: probe concentration is 10 μM; the concentration of agents 1–14 is 0.2 mM; the concentration of agents 15–39 is 1 mM; the anion for all cations is Cl^−^, except that NO_3_^−^ is for Ag^+^, Mn^2+^ and Pb^2+^. The cation for all anions is Na^+^. The PL emission at 503 nm and 615 nm was recorded under the excitation of 365 nm.

The PL curves of CSMPP in buffers with different pH in the range of pH 2.60–6.80 were measured. As shown in Fig. 2a, when the pH decreased from pH 6.80 to pH 2.60, the peak emission at 503 nm gradually weakened, and a new peak at 615 nm appeared and gradually enhanced. An isoemissive point at 560 nm was observed, indicating the conversion between two emissive species (non-protonated and protonated molecules). At the same time, the fluorescence images of the probe solution showed an obvious emission color change from green to yellow and then to red when the pH decreased from pH 6.80 to pH 3.45 (the inset pictures in Fig. 2b). In addition, the ratio of the PL emission at 615 nm to that at 503 nm (*I*_615_/*I*_503_) as a function of pH showed a reversed ‘S’ shape with a good fitting by the DoseResp function of the Origin software (Fig. 2b). After doing the calculation based on eqn (2) derived from Grynkiewicz's methods as shown in the ESI,† the log[(*R* − *R*_min_)/(*R*_max_ − *R*)] exhibited a good linear relationship with pH (Fig. 2c), suggesting that the ratiometric probe can be used for pH quantification. Moreover, the probe showed an absolute p*K*_a_ of 4.75 ± 0.02 (Fig. S6†), which is just fit in the lysosomal acidity window (4.5–5.5).^71–73^ Thus, CSMPP holds great promise to measure the lysosomal pH.

In addition, the absorption of CSMPP underwent a bathochromic shift with the absorption peak changing from 353 nm at pH 6.8 to 383 nm at pH 2.6 due to the molecular protonation (Fig. S7†). We guess that the large red-shifts in both the absorption (30 nm) and emission (112 nm) spectra were induced by the enhanced intramolecular charge transfer after the protonation of CSMPP.

### The mechanism of pH responsivity

To investigate the pH responsive mechanism of the CSMPP probe, the ^1^H NMR measurement and the density functional theory (DFT) calculation of the ground state of CSMPP and protonated CSMPP were carried out to check the protonation and the intramolecular charge transfer, respectively. The ^1^H NMR spectra of CSMPP in DMSO-*d*_6_ before and after addition of deuterium chloride were measured. As shown in Fig. S8,† the hydrogens in the methyl group at the end of the piperazinyl group and those in the pyridinyl group underwent an obvious down-shift, indicating that both the *N*-methyl-piperazinyl group and the pyridinyl group can be protonated. Since the p*K*_a_ of *N*-methylpiperidine and pyridine is 10.08 and 5.23 respectively,^74^ CSMPP has two groups of weak base, *i.e.* the *N*-methyl-piperazinyl group and the pyridinyl group, enabling the probe to target lysosomes with an acidic pH.

The results of DFT calculations showed that the electron clouds in the highest occupied molecular orbitals (HOMO) of CSMPP, CSMPP-H^+^ (protonated at the *N*-methyl-piperazinyl group) and CSMPP-2H^+^ (protonated at both the *N*-methyl-piperazinyl group and the pyridinyl group) were all essentially located around the cyanostylbene part (Fig. S9†). The electron clouds in the lowest unoccupied molecular orbitals (LUMO) of CSMPP and CSMPP-H^+^ were also mainly located around the cyanostylbene part, but that of CSMPP-2H^+^ was confined to the pyridium-phenyl part. In addition, the HOMO–LUMO energy gap of CSMPP-H^+^ showed a negligible change compared to the non-protonated CSMPP molecule. However, the HOMO–LUMO energy gap of CSMPP-2H^+^ (4.64 eV) was smaller than that of CSMPP (5.47 eV). These results suggest that the intramolecular charge transfer in the molecule becomes more obvious after the pyridinyl group being protonated, which can induce the redshift of both absorption and emission. Therefore, the enhanced intramolecular charge transfer induced by the protonation of the pyridinyl group in CSMPP forms the basis of the ratiometric fluorescence pH sensor.

### The reversibility and specificity of the pH probe

The pH reversibility of CSMPP was checked by adjusting the solution pH to pH 7 and pH 3 alternately (Fig. 2d). And it was found that the probe still functioned well even after undergoing five basic–acidic pH cycles. Since the selectivity is vital for the pH measurement both *in vitro* and *in vivo*, the interference of different chemical species with the proton binding by the probe was investigated. As shown in Fig. 2e, the emission ratio (*I*_615_/*I*_503_) at both pH 6.8 and pH 3.0 changed very little upon addition of 38 kinds of chemical species that exist in living systems, such as metal ions (K^+^, Na^+^, Ca^2+^, Mg^2+^, Cd^2+^, Co^2+^, Fe^2+^, Fe^3+^, Ni^2+^, Cu^2+^, Al^3+^, Zn^2+^, Ag^+^, Mn^2+^, and Pb^2+^), common anions (HPO_4_^2−^, H_2_PO_4_^−^, Ac^−^, NO_3_^−^, I^−^, and CO_3_^2−^), reactive oxygen species (ClO^−^ and H_2_O_2_), reactive sulphide species (HS^−^, SCN^−^, S_2_O_3_^2−^, cysteine, homocysteine, and glutathione), some natural amino acids and glucose. Therefore, the probe can be used for specific pH measurement without any interference from some common chemical species in living systems.

### Lysosome-specific imaging *in vitro* and the cytotoxicity of CSMPP

**Fig. 3:**
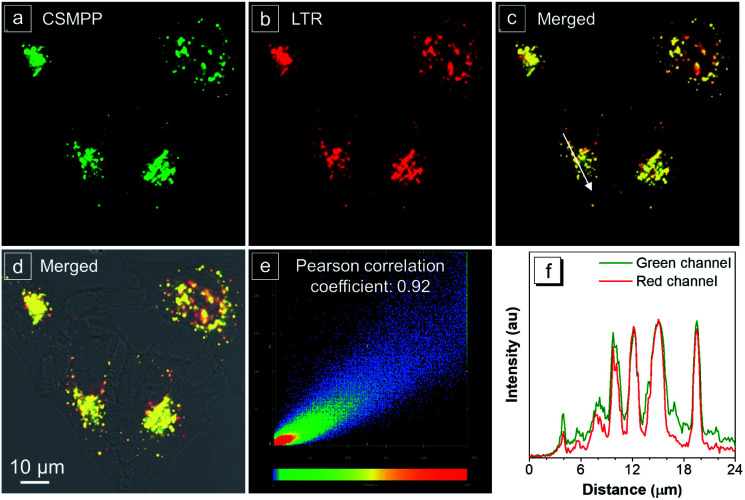
Confocal images of HeLa cells stained with 200 nM LysoTracker Red (LTR) for 10 min and then co-stained with 2 μM CSMPP for 10 min. Fluorescence images of (a) CSMPP; (b) LTR; (c) merged a and b; (d) merged brightfield with a and b; (e) the signal distribution diagram of the green channel for CSMPP and the red channel for LTR; (f) the fluorescence intensity profile extracted from the white arrow line in image c. Conditions: for CSMPP, *λ*_ex_ = 405 nm, *λ*_em_ = 450–580 nm. For LTR, *λ*_ex_ = 560 nm, *λ*_em_ = 650–700 nm. Scale bar is 10 μm.

HeLa cells are the most widely used cell model, and ARPE-19 cells, human retinal pigment epithelial cells, are often used as an *in vitro* model for age-related macular degeneration. Herein, the lysosome-specific staining by CSMPP *in vitro* was investigated by using HeLa cells and ARPE-19 cells, and captured with a confocal laser scanning microscope (CLSM). The results of the co-staining of HeLa cells with LysoTracker Red (LTR) and CSMPP showed that their emission channels overlapped well with a Pearson correlation coefficient of 0.92 (Fig. 3). And the co-staining of ARPE-19 cells with LysoTracker Blue (LTB) and CSMPP also showed a good Pearson correlation coefficient of 0.90 (Fig. S10†). Therefore, CSMPP can stain acidic organelles as the LysoTrackers do.

The cytotoxicity of CSMPP to HeLa cells and ARPE-19 cells was also investigated by using the MTT method. The MTT results showed that the viability of HeLa cells was kept above 91% (Fig. S11a†) and that of ARPE-19 cells was maintained above 80% (Fig. S11b†) when the CSMPP concentration was no more than 10 μM. Thus, the results indicate that CSMPP possesses low cytotoxicity.

### Lysosome-specific imaging *in vivo* and the biocompatibility of CSMPP

**Fig. 4:**
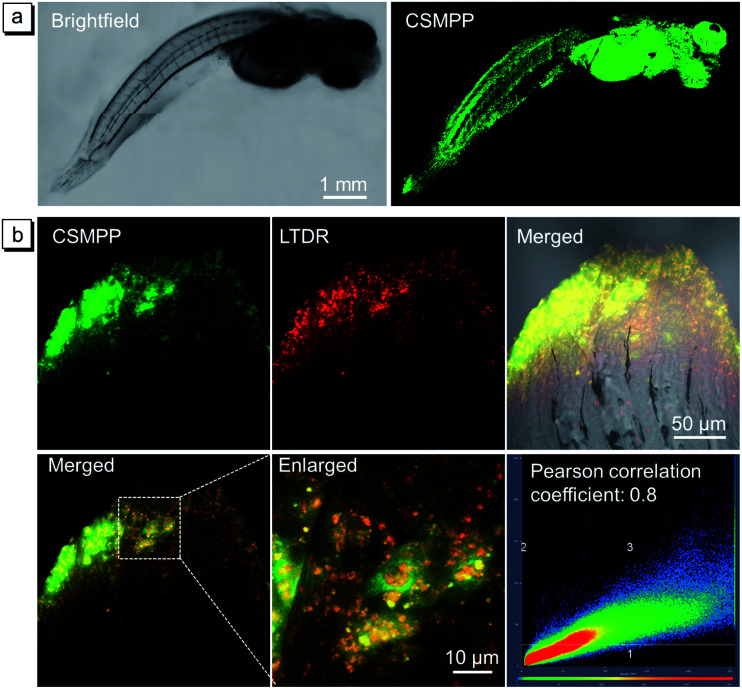
(a) Confocal images of the whole body of the medaka larva after being fed with CSMPP for 4 h. *λ*_ex_ = 405 nm, *λ*_em_ = 468–704 nm. Scale bar is 1 mm. (b) Confocal images of the caudal fin after co-staining of the medaka larva with CSMPP (5 μM) and LysoTracker deep red (LTDR, 200 nM). The images are the fluorescent pictures of CSMPP and LTDR, merged brightfield with them, merged two channels, enlarged image taken from the merged two channels, and the signal distribution diagram of the two channels. Conditions: for CSMPP, *λ*_ex_ = 405 nm, *λ*_em_ = 468–630 nm. For LTDR, *λ*_ex_ = 633 nm, *λ*_em_ = 647–713 nm. Scale bar is 50 μm. Scale bar for the enlarged image is 10 μm.

After the successful lysosome imaging *in vitro*, the lysosome-specific imaging of the medaka larvae's fin was investigated. After feeding the medaka larvae with 5 μM CSMPP for 4 h, the whole body of the medaka larva was lit up under light irradiation (Fig. 4a). Most importantly, the caudal fin was lit up. Then, we focused on the caudal fin to study the lysosome-specific imaging. After feeding the medaka larvae with CSMPP and LysoTracker deep red (LTDR), the confocal images of the caudal fin were captured (Fig. 4b). And the results showed that the signals of CSMPP and LTDR exhibited a good overlap with a Pearson correlation coefficient of 0.8 (Fig. 4b), indicating that CSMPP has good specificity to the lysosomes of the medaka larva's fin as LTDR does.

The biocompatibility of CSMPP to medaka larvae was evaluated using two methods. In the first method, medaka larvae were exposed to different concentrations of CSMPP (0–10 μM) for 96 h, and the survival rate was recorded (Fig. S12a†). The second method monitored the heartbeat rate of medaka larvae over 96 h after exposure to 5 μM CSMPP for 4 h (Fig. S12b†). The results showed that the survival rate was above 80% when the CSMPP concentration was no more than 10 μM (Fig. S12a†). And the heartbeat rate remained almost unchanged within 96 h after they were stopped from exposure to CSMPP (Fig. S12b†). Therefore, these results indicate that CSMPP has low toxicity towards medaka larvae.

### The photostability of CSMPP

**Fig. 5:**
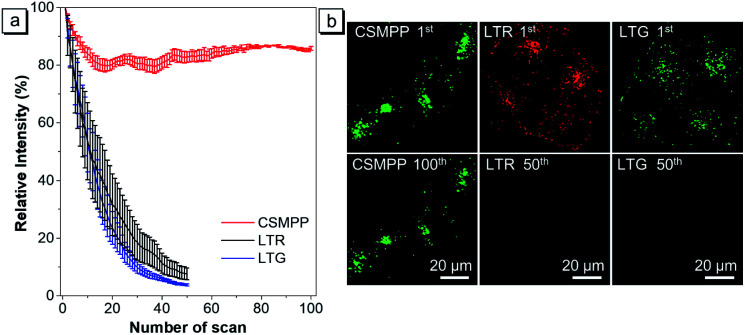
(a) Loss in fluorescence signals of CSMPP, LTR or LTG stained HeLa cells after continuous scanning using a CLSM. Data are the mean ± SD (*n* = 4). (b) Confocal images of HeLa cells stained with 2 μM CSMPP for 12 min at the 1^st^ and 100^th^ scans, 500 nM LTR for 6 min at the 1^st^ and 50^th^ scans, or 500 nM LTG for 5 min at the 1^st^ and 50^th^ scans. For CSMPP: *λ*_ex_ = 405 nm; *λ*_em_ = 470–650 nm. For LTR: *λ*_ex_ = 561 nm, *λ*_em_ = 565–650 nm. For LTG: *λ*_ex_ = 488 nm, *λ*_em_ = 495–580 nm. Scale bar is 20 μm.

For applications that require long-term tracking imaging, photostability is a critical requirement for fluorescence imaging. Therefore, the photostability of CSMPP was investigated by continuously scanning the stained HeLa cells 100 times (Fig. 5). As a comparison, the photostability of LTR and LysoTracker Green (LTG) was also measured. The results showed that after scanning 50 times, the fluorescence signals of LTR and LTG were almost reduced to zero (Fig. 5a and b), whereas more than 80% fluorescence signals of CSMPP were kept even after 100 sequential scans (Fig. 5a and b), suggesting the good photostability of CSMPP.

### Quantification of lysosomal pH *in vitro*

Before monitoring the dynamic lysosomal pH changes in living cells, the pH calibration curve of CSMPP in HeLa cells was established with the help of H^+^/K^+^ antiporter nigericin and H^+^/Na^+^ antiporter monensin to homogenize the pH inside and outside cells. The ratiometric image was acquired by dividing the fluorescence image of the red channel by that of the green channel (*E*_m-red_/*E*_m-green_). And the ratiometric images at different pH and the pH calibration curve are shown in Fig. S13.†

**Fig. 6:**
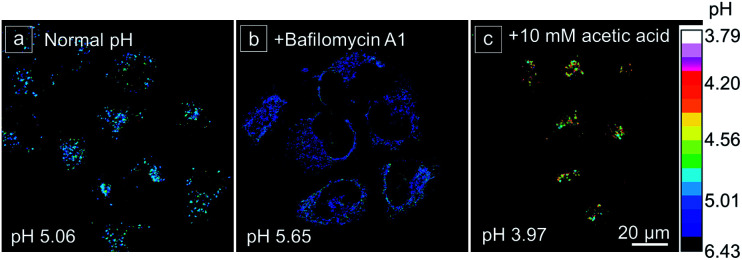
The ratiometric images of the lysosomal pH distribution of HeLa cells after being stimulated by chemical stimulants. (a) Normal lysosomal pH without stimulants; (b) 50 nM bafilomycin A1 incubated for 30 min; (c) 10 mM acetic acid incubated for 8 min at 37 °C. The captured confocal fluorescence images of red and green channels were analyzed using Image J software to acquire the ratiometric images. Scale bar is 20 μm.

To evaluate the performance of the lysosomal pH measurement using CSMPP, some common chemical stimulants were utilized to adjust the lysosomal pH (Fig. 6 and S14†). Then, the lysosomal pH was measured based on the pH calibration curve acquired in HeLa cells. Firstly, the normal lysosomal pH was measured, giving a pH value of 5.06 (Fig. 6a). It should be noted that the measured normal lysosomal pH is similar to that measured by other reported probes.^75^ It is well known that bafilomycin A1 can increase the lysosomal pH by inhibiting the vacuolar-type H^+^-ATPase (proton pump) from protonating lysosomes. When the probe-stained HeLa cells were further incubated with 50 nM bafilomycin A1 for 30 min, the lysosomal pH was increased to 5.65 (Fig. 6b). Acetic acid is a weak organic acid that can freely diffuse into cells.^76^ When HeLa cells were incubated with 10 mM acetic acid for 8 min, the lysosomal pH decreased to 3.97 (Fig. 6c). Moreover, it showed a concentration-dependent acidification of the cells, and the lysosomal pH decreased to 4.45 after incubation with 1.75 mM acetic acid for 8 min (Fig. S14†). H_2_O_2_ is a kind of reactive oxygen species, and it can exert oxidative stress on cells and impair the vacuolar-type H^+^-ATPase, resulting in the alkalization of lysosomes.^77^ When HeLa cells were treated with 0.1 mM H_2_O_2_ for 30 min, the lysosomal pH increased to 5.55 (Fig. S14†). Since tamoxifen is known to alkalinize lysosomal pH,^78^ it increased the lysosomal pH to 6.14 after cell incubation with 40 μM tamoxifen for 15 min at 37 °C (Fig. S14†). In addition, we found that when the CSMPP incubation concentration was changed, the lysosomal pH of HeLa cells showed no significant change (Fig. S15†), which is because ratiometric imaging was not affected by the probe concentration. Therefore, these results suggest that the CSMPP probe can sensitively monitor and quantify lysosomal pH in living cells.

### *In vivo* lysosomal pH tracking during the caudal fin regeneration

**Fig. 7:**
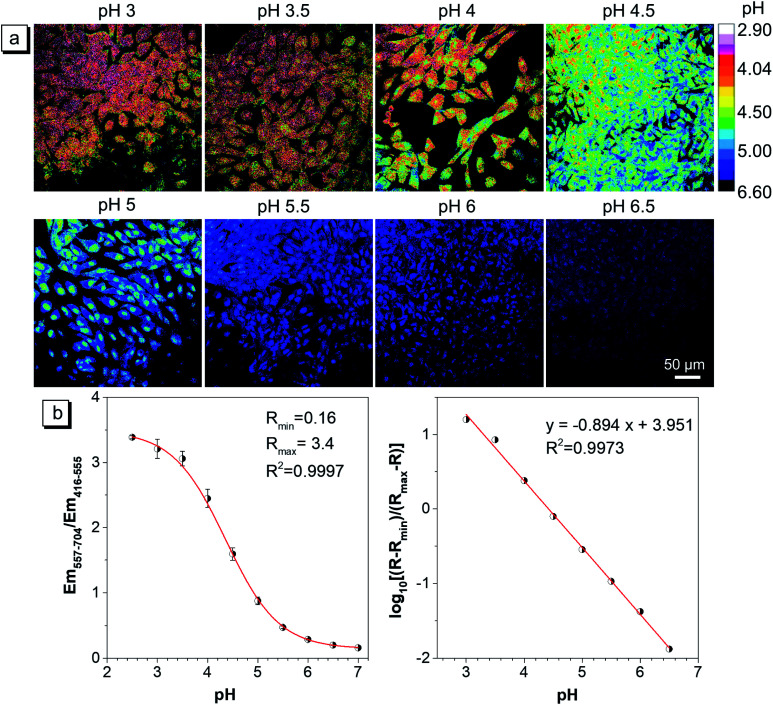
(a) The pH calibration of the CSMPP probe in zebrafish cells captured using a CLSM. Cells were first stained with 3 μM CSMPP for 1 h at 37 °C. Then, the cells were equilibrated in pH calibration buffers containing 12 μM nigericin and 5 μM monensin for 8 min at 25 °C. For the green channel: *λ*_em_ = 416–555 nm; for the red channel: *λ*_em_ = 557–704 nm. *λ*_ex_ = 405 nm. Scale bar is 50 μm. (b) The mean ratio of *E*_m-red_/*E*_m-green_ as a function of pH. The mean ± SD between three images was presented. The calibration curve was fitted based on the relationship among the mean ratio, *R*_min_, *R*_max_ and the pH, whose calculation equation is presented in the ESI.†

Since the capture conditions of the caudal fin were different from those of HeLa cells, it is necessary to conduct the pH calibration experiment again. It was carried out by using fish cells and a 40× oil objective lens under the same capture conditions as those of the caudal fin. The ratiometric images (*E*_m-red_/*E*_m-green_) at different pH and the pH calibration curve are exhibited in Fig. 7a and b, respectively. The results showed that with the increase of buffer pH, the mean ratio exhibited a reversed ‘S’ curve as a function of pH, and the *R*_min_ and *R*_max_ were also fitted (Fig. 7b). The log[(*R* − *R*_min_)/(*R*_max_ − *R*)] and pH showed a good linear relationship in the pH range of 3.0–6.5 (Fig. 7b), which will be used to calibrate the lysosomal pH of the caudal fin.

**Fig. 8:**
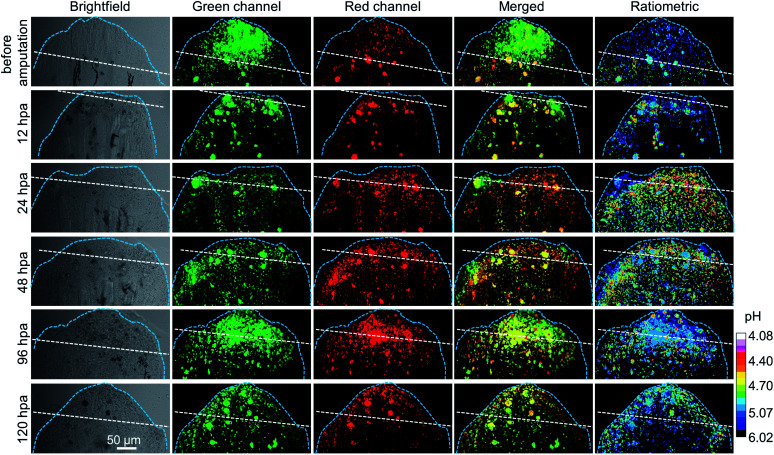
The CSLM images of the medaka larva's caudal fin before amputation and after amputation at different times (12, 24, 48, 96 and 120 hpa), including brightfield, green channels, red channels, merged two-channel images, and ratiometric images showing lysosomal pH distribution. For the green channel: *λ*_em_ = 416–555 nm; for the red channel: *λ*_em_ = 557–704 nm. *λ*_ex_ = 405 nm. The ratiometric images of *E*_m-red_/*E*_m-green_ were analyzed by using Image J software based on the confocal fluorescence images. Hours post-amputation is short for hpa. White dashed line indicates the amputation plane. Light blue dashed line indicates the outline of the caudal fin. Scale bar is 50 μm.

Then, the lysosomal pH tracking during the caudal fin regeneration of the medaka larva was performed. The medaka larvae were first fed with 5 μM CSMPP for 4 h, and then their caudal fins were amputated. The regeneration of the caudal fin of the medaka larva was tracked by capturing it with a CSLM before amputation and at 12, 24, 48, 96 and 120 hours post-amputation (hpa). As shown in Fig. 8, at 12 hpa, the end of the caudal fin with an originally round shape became truncated after being amputated by a razor blade. Then, the caudal fin regrew gradually. At 120 hpa, the end of the caudal fin became nearly round and the fin almost reached the original length, which indicates that the amputated caudal fin was about to be completely regenerated into the original one.

**Fig. 9:**
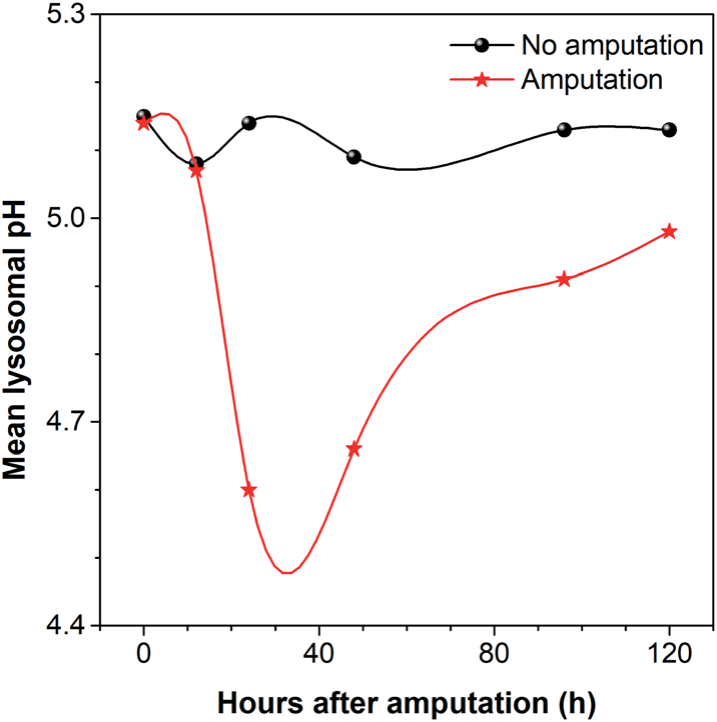
The mean lysosomal pH change of the medaka larva's caudal fin during regeneration. As a comparison with the amputated medaka larva, the medaka larva without amputation after being fed with CSMPP was photographed at the same time as that of photographing the amputated ones. The hours shown here were named based on the hours after amputation. And “0 h” means the time after being fed with CSMPP and before amputation.

During the regeneration process, the lysosomal pH of the caudal fin changed a lot. As shown in Fig. 9, the mean lysosomal pH gradually decreased after amputation, and the lowest pH was reached during 24–48 hpa, which is reported to be the stage of blastema formation.^79^ And the mean lysosomal pH reduced by 0.5 at 24 hpa, changing from original pH 5.1 to pH 4.6. This indicates that lysosomes were the most active at this stage. This is because regeneration relies heavily on autophagy to clear the damaged tissue and cellular debris resulting from injury.^6^ Thus, to aid the autophagy for degradation, the lysosome acidification of cells was upregulated. In addition, it was observed that the lysosomes around the amputation plane (including the regenerated part) were more acidic than those distributed far away from the amputation plane at 24 and 48 hpa (Fig. 8). Nevertheless, the lysosomal pH increased after 48 hpa and returned to normal after 120 hpa (Fig. 9). As a comparison, the caudal fin of the medaka larva without amputation showed almost no change in the lysosomal pH during five-days of tracking (Fig. 9 and S16†).

Since some studies have found that autophagy is activated, V-ATPase expression and lysosomal acidification are upregulated during tissue regeneration,^6,7,80–83^ and the observed phenomenon of lysosomal acidification in our work is consistent with the reported results. But our research provides a simpler method and shows a more detailed change in the mean lysosomal pH and its distribution during caudal fin regeneration.

## Conclusions

In summary, we have developed a lysosomal pH probe with AIE properties through a simple two-step synthesis. The CSMPP probe exhibited a pH-dependent emission at 503 nm and 615 nm with a red-shift of 112 nm due to the greatly enhanced intramolecular charge transfer, thus allowing the ratiometric measurement of lysosomal pH. Moreover, the CSMPP probe showed good specificity to protons, reversibility to pH responses, lysosome-targeting ability, good biocompatibility, excellent photostability, the ability to sensitively quantify lysosomal pH both *in vitro* and *in vivo*, and the ability to track lysosomal pH in the long term. In addition, our study has successfully achieved the *in vivo* dynamic monitoring of the process of tissue regeneration for the first time. Our probe monitored the changes in lysosomal pH during the medaka fin regeneration until the caudal fin almost regenerated to the original length and shape. It was found that during the fin regeneration, lysosomal acidification is required to facilitate the elevated level of autophagic activity in the cells around the amputation plane (including the regenerated part) after amputation for 24–48 h. The mean lysosomal pH was reduced by 0.5 at 24 hpa and then returned to normal pH at 120 hpa. Therefore, our AIE probe quantified the detailed changes in the mean lysosomal pH and exhibited its distribution during the caudal fin regeneration. Moreover, our AIE probe provides a simple and quantitative method for dynamically tracking tissue regeneration. We believe that the lysosomal pH probe can be used in tracking various lysosome-involved biological processes in the future, such as tracking the stress responses of tissue, tracking the inflammatory responses, and so on. This work can further inspire the design of other ratiometric fluorescent probes for the long-term visualized tracking of a wide variety of important biological processes.

## Experimental procedures

### Materials and instruments

4-(4-Methylpiperazin-1-yl)benzaldehyde (TCI America), 2-(4-bromophenyl)acetonitrile (J&K), pyridin-4-ylboronic acid (J&K), tetrakis(triphenylphosphine)palladium(0) (Energy), ethanol (AR, J&K), dichloromethane (AR, J&K), 4-morpholineethanesulfonic acid (Sigma-Aldrich), HEPES (Sigma-Aldrich), sodium acetate (Sigma-Aldrich) and acetic acid (Glacial, VWR Chemicals) were used directly as received. LysoTracker probes (Invitrogen/Molecular Probes) were used to label lysosomes in live cells as described in the protocols. Nigericin sodium salt (Sigma-Aldrich) was dissolved in ethanol as the stock solution. Monensin sodium salt, bafilomycin A_1_, tamoxifen and chloroquine were from Sigma-Aldrich, and dissolved in DMSO as stock solutions. Milli-Q water was purified *via* the Milli-Q Plus System (Millipore Corporation, United states). All other reagents were commercially available and used as supplied without further purification. Compound **3** was prepared according to our published paper.^84^

The ^1^H NMR and ^13^C NMR spectra were recorded using a Bruker AVII 400 MHz NMR spectrometer, and samples were prepared with deuterated solvent. High-resolution mass spectra were obtained on a Finnigan MAT TSQ 7000 Mass Spectrometer operated in the MALDI-TOF mode. UV-vis absorption and photoluminescence (PL) spectra were recorded on a Shimadzu 2550 UV/VIS spectrophotometer and Horiba Fluorolog-3 spectrofluorometer, respectively. The absolute fluorescence quantum yields were measured on a Hamamatsu Absolute Quantum Yield Spectrometer C13534. The average particle size and size distribution of the samples were determined using a Brookhaven ZetaPlus potential analyzer (Brookhaven instruments corporation, USA). Single crystal X-ray diffraction data were collected on an Oxford Diffraction Xcalibur Atlas Gemini ultra-instrument. The electron density contours of the HOMOs and LUMOs based on Frontier molecular orbitals at S0 for CSMPP before and after acidification were simulated by Gauss View software, which was optimized by the M062X/6-31G(d,p) level. The pH was measured using an ION 700 Benchtop Meter (Oakton).

### Synthesis of CSMPP

The synthetic route is shown in Scheme S1.†**3** (0.1 g, 0.515 mmol) and **4** (0.105 g, 0.515 mmol) in 4 mL of ethanol were dissolved in a 50 mL round bottom flask. Sodium hydroxide (20.6 mg, 0.515 mmol) was dissolved in 1 mL ethanol, and then added slowly into the mixture. After stirring for 2 h, the pale-yellow precipitates were filtered, washed with cold ethanol and dried under reduced pressure. Yield: 80%. The crystal of CSMPP was acquired by slow evaporation of its nearly saturated solution in chloroform. ^1^H NMR (400 MHz, CDCl_3_), *δ* (ppm): 8.76–8.60 (m, 2H), 7.90–7.83 (m, 2H), 7.74 (d, *J* = 8.6 Hz, 2H), 7.68 (d, *J* = 8.5 Hz, 2H), 7.55–7.49 (m, 2H), 7.47 (s, 1H), 7.01–6.84 (m, 2H), 3.45–3.29 (m, 4H), 2.56 (t, *J* = 5.1 Hz, 4H), 2.35 (s, 3H). ^13^C NMR (400 MHz, CDCl_3_), *δ* (ppm): *δ* 152.47, 150.33, 147.25, 142.52, 137.73, 136.05, 131.32, 127.46, 126.19, 123.69, 121.36, 118.82, 114.36, 105.32, 54.73, 47.36, 46.13. HRMS (MALDI-TOF): *m*/*z* 381.2085 (M^+^, calcd 380.2001).

### pH responsivity and reversibility

The HEPES/MES/acetate buffers containing 20 mM HEPES, 20 mM MES, 20 mM acetate, 100 mM KCl and 20 mM NaCl were adjusted to different pH by using 2 N NaOH solution and 2 N hydrochloric acid. The PL spectra of 10 μM CSMPP (containing 2% DMSO) in buffers in the pH range of 2.60–6.80 were recorded with an excitation of 365 nm. The pictures of CSMPP in different pH buffers were taken using a digital camera under a hand-held UV lamp with an excitation of 365 nm. The UV-vis absorption spectra of 10 μM CSMPP (containing 2% DMSO) in buffers at pH 2.60 and 6.80 were measured.

The reversibility of pH responses to pH 7 and pH 3 of 10 μM CSMPP solution (containing 2% DMSO) was checked by continuously adjusting the pH from 7 to 3 with concentrated hydrochloric acid and 10 N NaOH solution for 5 cycles. Fluorescence was excited at 365 nm. The PL intensities at 503 and 615 nm were recorded.

### Interference in pH measurements

The interference of different chemical species with probe fluorescence in HEPES/MES/acetate buffers at pH 6.8 and pH 3.0 was checked. To the solutions of 10 μM CSMPP in buffers at pH 6.8 and pH 3.0 (containing 2% DMSO), 0.2 mM CaCl_2_, 0.2 mM MgCl_2_, 0.2 mM CdCl_2_, 0.2 mM CoCl_2_, 0.2 mM FeCl_2_, 0.2 mM FeCl_3_, 0.2 mM NiCl_2_, 0.2 mM CuCl_2_, 0.2 mM AlCl_3_, 0.2 mM ZnCl_2_, 0.2 mM AgNO_3_, 0.2 mM Mn(NO_3_)_2_, 0.2 mM Pb(NO_3_)_2_, 1 mM KCl, 1 mM NaCl, 1 mM NaI, 1 mM Na_2_HPO4, 1 mM NaH_2_PO_4_, 1 mM NaAc, 1 mM NaNO_3_, 1 mM Na_2_CO_3_, 1 mM NaClO, 1 mM NaHS, 1 mM NaSCN, 1 mM Na_2_S_2_O_3_, 1 mM H_2_O_2_, 1 mM tryptophan, 1 mM glutamine, 1 mM aspartic acid, 1 mM glycine, 1 mM leucine, 1 mM valine, 1 mM cysteine, 1 mM homocysteine, 1 mM glutathione, 1 mM arginine, 1 mM serine and 1 mM glucose were added, respectively. Then, the PL intensities at 503 and 615 nm before and after addition of chemical species were recorded under the excitation of 365 nm.

### Cell culture

All cells were purchased from ATCC. HeLa cells were cultured in Dulbecco's modified Eagle's medium (DMEM, Gibco) supplemented with 10% heat-inactivated fetal calf serum (FCS, Gibco) and 50 μg mL^−1^ penicillin/streptomycin (Hyclone) at 37 °C in a humidified incubator containing 5% CO_2_. ARPE-19 cells (retinal pigmented epithelium) were cultured in DMEM: F-12 Medium (Life Technologies, Gaithersburg, MD) with 10% heat-inactivated fetal calf serum (FCS, Gibco) and 50 μg mL^−1^ penicillin/streptomycin (Hyclone) at 37 °C in a humidified incubator containing 5% CO_2_. Zebrafish cells (ZF4 cell line) derived from 1 day old zebrafish embryos were maintained in DMEM medium supplemented with 10% (v/v) FBS, 20 IU mL^−1^ penicillin and 20 mg L^−1^ streptomycin. The cells were sub-cultured by treating with 0.25% (w/v) trypsin–0.53 mM EDTA solution after they reached 95% confluence.

### Lysosomal pH measurement of cells after being stimulated

To study the influence of stimulants on the lysosomal pH of HeLa cells, the cells were first loaded with 5 μM CSMPP for 15 min at 37 °C, followed by PBS washing and 30 min of internalization, and then the stained cells were treated with 50 nM bafilomycin A1 for 30 min, 0.1 mM H_2_O_2_ for 30 min, 40 μM tamoxifen for 15 min, and 1.75 mM and 10 mM acetic acid for 8 min at 37 °C. The confocal images with two channels were captured using a CLSM with a 63× oil objective lens and an excitation of 405 nm. The fluorescence emission of the green channel at 470−560 nm and red channel at 560−700 nm was collected. The same settings (*e.g.* objective lens, channel range, pinhole, laser gain, and resolution) as those used in performing pH calibration experiments for HeLa cells were employed during lysosomal pH measurements. Image J software was used to analyze the fluorescence images. After performing background deduction, the ratio of the red channel to the green channel was calculated. And the mean lysosomal pH was calculated based on the pH calibration curve for HeLa cells.

### Culture of medaka and medaka larvae

Japanese medaka (Oryzias melastigma (O. melastigma)) fishes were cultured based on the OECD guidelines (OECD Test Guidelines 203 and 210). Medaka fishes were cultured in a 40 L water tank with filtered creek water (GF/C membrane with a pore size of 1.2 μm, Millipore, Watford, U.K.), which was collected from the campus pond of the Hong Kong University of Science and Technology. The medaka fishes were fed with newly hatched brine shrimp (Artemia salina) twice a day. The photoperiod (16 h light/8 h dark) was maintained at a constant temperature (28 °C). The females spawned every day, and the fertilized egg clusters were collected from the belly of a female and rinsed with DI water to remove the sludge and to separate the clusters into single eggs. The eggs were then rinsed and placed in ERM (embryo rearing medium), which was prepared by dissolving 1 g NaCl, 0.03 g KCl, 0.04 g CaCl_2_·2H_2_O and 0.163 g MgSO_4_·7H_2_O in 1 L ultrapure water, adjusted to pH 7.2 with 1.25% NaHCO_3_ solution and filtered to sterilize. 1 day old medaka larvae were used in this study for lysosome tracking because they have a transparent body at the early stage of life.

### Long-term tracking lysosomal pH during medaka fin regeneration

Medaka larvae were placed in a glass beaker containing 100 mL of ERM and 5 μM CSMPP for 4 h at 28 °C. The medaka larvae were transferred to new ERM, and then anesthetized with 0.005% tricaine in a glass-bottomed Petri dish. Confocal images were taken using a Zeiss LSM 710 confocal microscope with a 40× oil objective lens, an excitation of 405 nm and two channels. The green channel had an emission range of 416−555 nm; the red channel had an emission range of 557−704 nm. The same settings (*e.g.* objective lens, channel range, pinhole, laser gain, and resolution) as those used in performing pH calibration experiments for zebrafish cells were employed during lysosomal pH measurements.

After taking the confocal images of the caudal fin of the medaka larva before amputation, the medaka larva was anesthetized in 0.6 mM tricaine and carefully amputated using a razor blade. Then, the amputated medaka larva continued to be cultured in ERM at 28 °C. At different time points, which were expressed as hours post-amputation (hpa), *i.e.* 12 hpa, 24 hpa, 48 hpa, 96 hpa and 120 hpa, the confocal images of the caudal fin of the medaka larva were captured. The confocal images of the medaka larvae without incubation with CSMPP were also captured under the same imaging parameters, which were used as background signals. Image J software was used to analyze the fluorescence images. After performing background deduction, the ratio of the red channel to the green channel was calculated. And the mean lysosomal pH was calculated based on the pH calibration curve acquired in zebrafish cells. The mean lysosomal pH was calculated from the lysosomes of the caudal fin over a length of about 170 μm from the end of the caudal fin (including the regenerated part). Three independent experiments were carried out, and similar results were obtained.

As a comparison to the medaka larvae with amputation, the lysosomal pH of the caudal fin of control medaka larvae without amputation was also monitored by using a CLSM. And the medaka larvae without amputation after being fed with CSMPP were photographed at the same time as that of photographing the amputated ones. The hours used here were named based on the hours after amputation. And “0 h” means the time after being fed with CSMPP and before amputation.

## Ethical statement

All experiments were performed in accordance with the Guidelines of Animal and Plant Care Facility. And experiments were approved by the ethics committee at the Hong Kong University of Science and Technology. Informed consent was obtained from human participants of this study.

## Conflicts of interest

There are no conflicts of interest to declare.

## Supplementary Material

SC-011-C9SC06226B-s001

SC-011-C9SC06226B-s002
