# Supplementary material for: In vivo monitoring of tissue regeneration using a ratiometric lysosomal AIE probe
Source: Chem Sci. 2020 Feb 11;11(12):3152–63. doi: 10.1039/c9sc06226b (PMC8157324; doi:10.1039/c9sc06226b)
Supplement: SC-011-C9SC06226B-s001 [file SC-011-C9SC06226B-s001.pdf]

Electronic Supplementary Material (ESI) for Chemical Science.
This journal is © The Royal Society of Chemistry 2020

Electronic Supplementary Information

In vivo monitoring tissue regeneration by a ratiometric lysosomal AIE probe†

Xiujuan Shi‡,[a,b] Neng Yan‡,[c] Guangle Niu,[a,b] Simon H. P. Sung,[b] Zhiyang Liu,[b] Junkai Liu,[b] Ryan T. K. Kwok,*[,a,b] Jacky W. Y. Lam,[b] Wen-Xiong Wang,*[,a,d] Herman H.-Y. Sung,[b] Ian D. Williams[b] and Ben Zhong Tang*[,a,b,e,f]

a. HKUST-Shenzhen Research Institute, No. 9 Yuexing 1st RD, South Area, Hi-tech Park, Nanshan, Shenzhen 518057, China.

b. Department of Chemical and Biological Engineering, Department of Chemistry, Hong Kong Branch of Chinese National Engineering Research Center for Tissue Restoration and Reconstruction, Institute for Advanced Study, The Hong Kong University of Science and Technology, Clear Water Bay, Kowloon, Hong Kong, China.

c. Department of Ocean Science, The Hong Kong University of Science and Technology, Clear Water Bay, Kowloon, Hong Kong, China.

d. School of Energy and Environment, State Key Laboratory of Marine Pollution, City University of Hong Kong, Kowloon, Hong Kong, China.

e. Ming Wai Lau Centre for Reparative Medicine, Karolinska Institutet, Hong Kong, China.

f. Centre for Aggregation-Induced emission, SCUT-HKUST Joint Research Laboratory, State Key Laboratory of Luminescent Materials and Devices, South China University of Technology, Guangzhou 510640, China.

1 Experimental procedures

1.1 Colocalization with commercial LysoTrackers in both mammal cells and medaka larvae

To investigate the specificity of lysosome targeting by CSMPP probe, the *in vitro* co-localization studies were performed with HeLa cells and ARPE-19 cells. HeLa cells were incubated with 200 nM LysoTracker Red (LTR, Invitrogen Co., Carlsbad, CA) for 10 min and then co-stained with 2 μM CSMPP for 10 min at 37 °C. For CSMPP, λ_{ex} = 405 nm, λ_{em} = 450–580 nm. For LTR, λ_{ex} = 560 nm, λ_{em} = 650–700 nm. ARPE-19 cells were incubated with 240 nM LysoTracker Blue (LTB, Invitrogen Co., Carlsbad, CA) for 10 min and then co-stained with 1.5 μM CSMPP for 10 min. For CSMPP, λ_{ex} = 405 nm, λ_{em} = 480–700 nm. For LTB, λ_{ex} = 405 nm, λ_{em} = 400–440 nm.

The *in vivo* co-localization was also studied. Medaka larvae were firstly exposed to 5 μM CSMPP for 2 h at 28 °C, and then were fed with 200 nM LysoTracker deep red (LTDR, Invitrogen Co., Carlsbad, CA) together with CSMPP for another 2 h at 28 °C. After being washed with ERM, medaka larvae were imaged under two different channels. Green channel was excited at 405 nm and detected from 468 to 630 nm; red channel was excited at 633 nm and detected from 647 to 713 nm. These images were obtained by using a Zeiss LSM 710 confocal microscope equipped with the accessory of a Bioptechs Focht Chamber System 2 (FCS 2) and analyzed by using ZEN 2009 software (Carl Zeiss).

1.2 Cytotoxicity study

2-(4,5-Dimethyl-2-thiazolyl)-2,5-diphenyltetrazolium bromide (MTT) assay was used to evaluate the cytotoxicity of CSMPP to HeLa cells and ARPE-19 cells. HeLa cells or ARPE-19 cells were seeded in a 96-well plate at a density of 5,000 cells per well. After 24 h incubation, the cells were exposed to a series of doses of CSMPP (0–10 μM) in culture medium at 37 °C for 24 h. Six replicates were performed for each concentration. Next, the dye solution in wells were removed and 0.5 mg/mL freshly prepared MTT solution was added into each well. After incubation for 4 h, 100 μL of solubilization solution (10% SDS in 0.01 M HCl) was added to dissolve the formed purple crystals. After another 4 hours' incubation, the absorbance at 570 nm was recorded using a Perkin-Elmer Victor plate reader.

1.3 Intracellular pH calibration and measurement

The pH calibration buffer contains 20 mM HEPES, 20 mM MES, 20 mM acetate, 100 mM KCl, 20 mM NaCl, 1 mM $CaCl_2$, 0.5 mM $MgCl_2$, 5 mM glucose, 12 μM nigericin and 5 μM monensin. The pH buffers were adjusted to different pH in the range of 2.50–7.00 by using 2 N NaOH solution and 2 N hydrochloric acid.

HeLa cells or zebrafish cells were cultured in 35 mm confocal dishes (VWR International) and used when they were grown up to about 80% confluence. Cells were first stained by 3 μM CSMPP for 1 h at 37 ºC. Then, cells were equilibrated in pH calibration buffers for 8 min at 25 ºC. The fluorescence images of green channel and red channel at four fields of vision for each pH were acquired in less than 30 min. The fluorescence images for pH calibration in HeLa cells were captured by CLSM with a 63x oil objective lens. The fluorescence images for pH calibration in zebrafish cells were captured by CLSM with a 40x oil objective lens. Confocal images of cells without incubation by dyes were also captured and analyzed as the background signals, which will be used as the minimal threshold of the fluorescence images for pH calibration.

The ratiometric analysis was carried out by using Image J software. The background of every fluorescence image was subtracted by using the minimal threshold after transforming the image into 32-bit image. For HeLa cells captured by 63x oil objective lens, the minimal threshold is 5. For zebrafish cells captured by 40x oil objective lens, the minimal threshold is 2. The ratiometric image was acquired by dividing the red image by the green image with Image J software. From the ratiometric image, the mean lysosomal pH was calculated. The ratiometric image was shown in 16-color type under the lookup tables. The same settings (e.g. objective lens, channel range, pinhole, laser gain, resolution) as those used in performing pH calibration experiments were employed for the imaging of corresponding cells during lysosomal pH measurement.

The H^+ calibration equation was acquired based on Grynkiewicz's formula for calibration of calcium ion,[1] assuming that the fluorescence contribution from any given molecular species is proportional to the concentration of that species.

Therefore, the relationship between $[H^+]$ and ratio is shown as below:

$$[H^+] = K_a \left[\frac{R-R_{min}}{R_{max}-R}\right] \left[\frac{S_{f2}}{S_{b2}}\right] \quad (1)$$

The fluorescence ratio R is the ratio of the dye's fluorescence intensities F_1 and F_2 at two emission wavelengths λ_1 and λ_2 ($R = F_1/F_2$). K_a is the acid dissociation constant ($K_a = \frac{[H^+][A^-]}{[HA]}$). S factor is the proportionality coefficient in relation with dye concentration, and S_{f2} is symbolized for free dye measured at wavelength λ_2, S_{b2} for H^+-bound dye at λ_2. It is well known that, in principle, each S factor is determined by the excitation intensity, extinction coefficient, path length, quantum efficiency and the instrumental efficiency of collecting emitted photons.

Taking the equation $pH = -\log[H^+]$ and $pK_a = -\log K_a$ into equation (1), the linear relationship between pH and $\log[\frac{R-R_{min}}{R_{max}-R}]$ was acquired as below:

$$pH = -\log\left[\frac{R-R_{min}}{R_{max}-R}\right] + pK_a - \log\left[\frac{S_{f2}}{S_{b2}}\right] \quad (2)$$

After performing the pH calibration experiments, the linear fitting relationship between pH and $\log[\frac{R-R_{min}}{R_{max}-R}]$ under the specific imaging conditions can be obtained based on the equation (2). Then,

the measured mean ratio R of samples was taken into the fitted linear calibration curve to calculate the corresponding pH.

1.4 *In vivo* toxicity to medaka

The biocompatibility of CSMPP was assessed by exposing medaka larvae to different concentrations of CSMPP (0−10 μM) in a glass beaker containing 1 L of ERM for 96 h at 28 °C. In each beaker, 10 individuals of 1-day old medaka larvae were added. Three replicates were performed for each treatment. All the beakers were stored in an incubator at 28 °C. Mortality of medaka larvae was then monitored for 96 h. In addition, the biocompatibility of CSMPP was also evaluated by recording the heartbeat of larvae. Fifteen medaka larvae were first incubated with 5 μM CSMPP for 4 h, then transferred into new ERM, later the heartbeat of the larvae was recorded at different time (0, 6, 24, 48, 72 and 96 h). Heart rate was measured as mean heartbeat per minute. Larvae were allowed to “rest” for roughly one minute before being measured. A hand-held counter was used to record heartbeats for 15 seconds.

1.5 Photostability study

HeLa cells were stained by 2 μM CSMPP for 12 min, 500 nM LTR for 6 min and 500 nM LTG for 5 min at 37 °C, respectively. Then, the confocal fluorescence images of the CSMPP-stained HeLa cells were continuously scanned for 100 times using its normal imaging condition. The confocal fluorescence images of the LTR-stained or LTG-stained HeLa cells were continuously scanned for 50 times using their normal imaging conditions. And the changes of fluorescence signals were compared. For CSMPP: λ_{ex} = 405 nm; λ_{em} = 470–650 nm. For LTR: λ_{ex} = 561 nm, λ_{em} = 565–650 nm. For LTG: λ_{ex} = 488 nm, λ_{em} = 495–580 nm.

2 Results

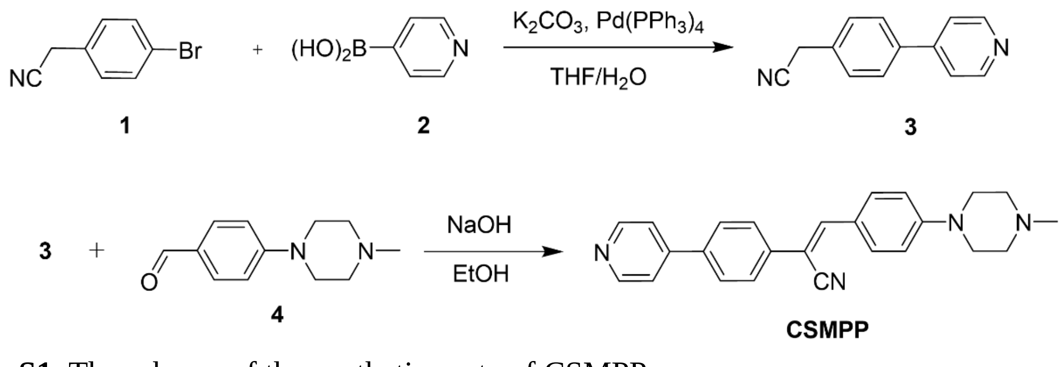

Scheme S1. The scheme of the synthetic route of CSMPP.

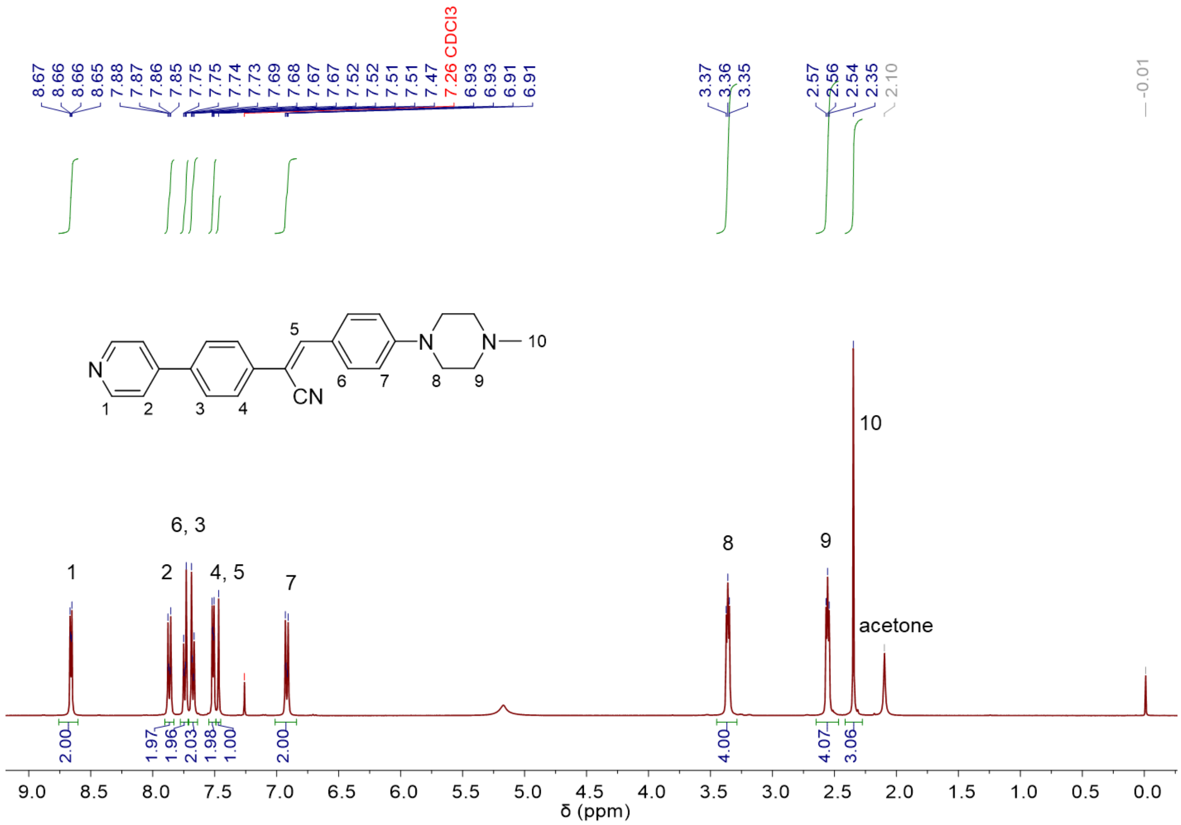

Figure S1. The ^{1}H NMR spectrum of CSMPP in $CDCl_3$.

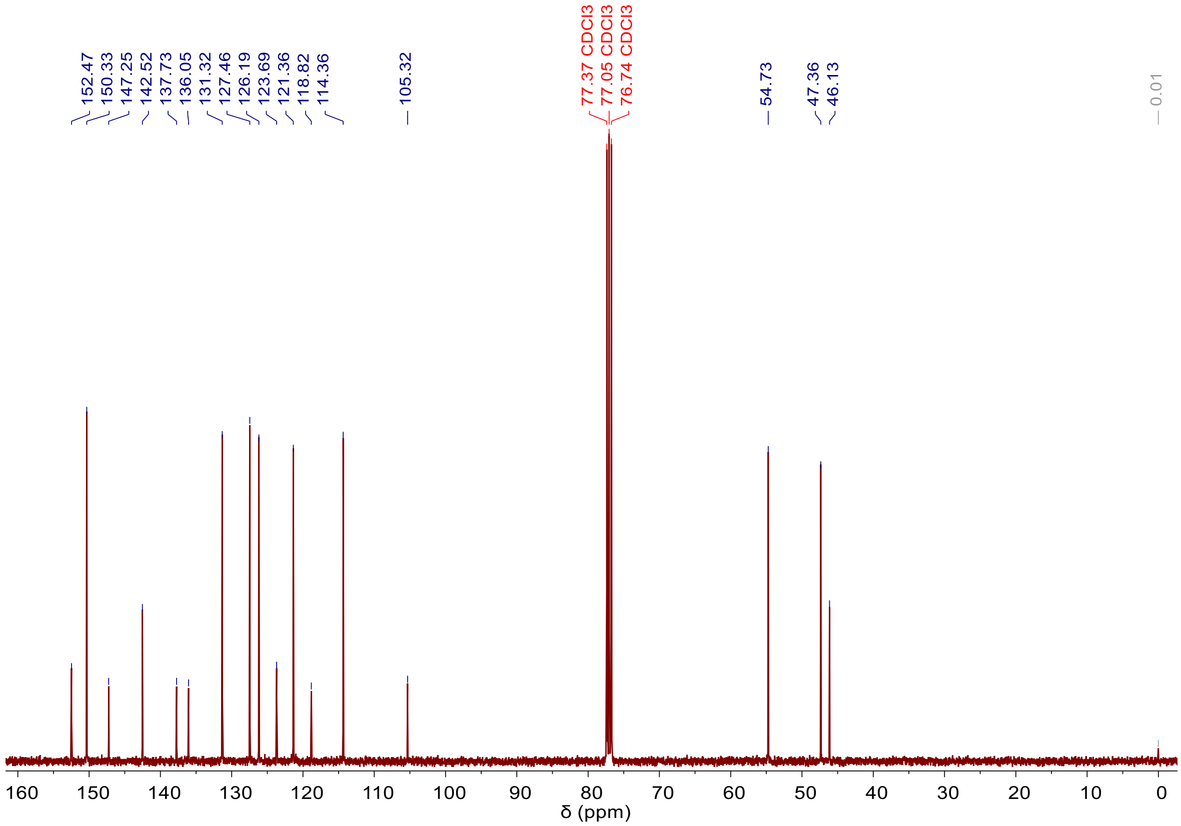

Figure S2. The ^{13}C NMR spectrum of CSMPP in $CDCl_3$.

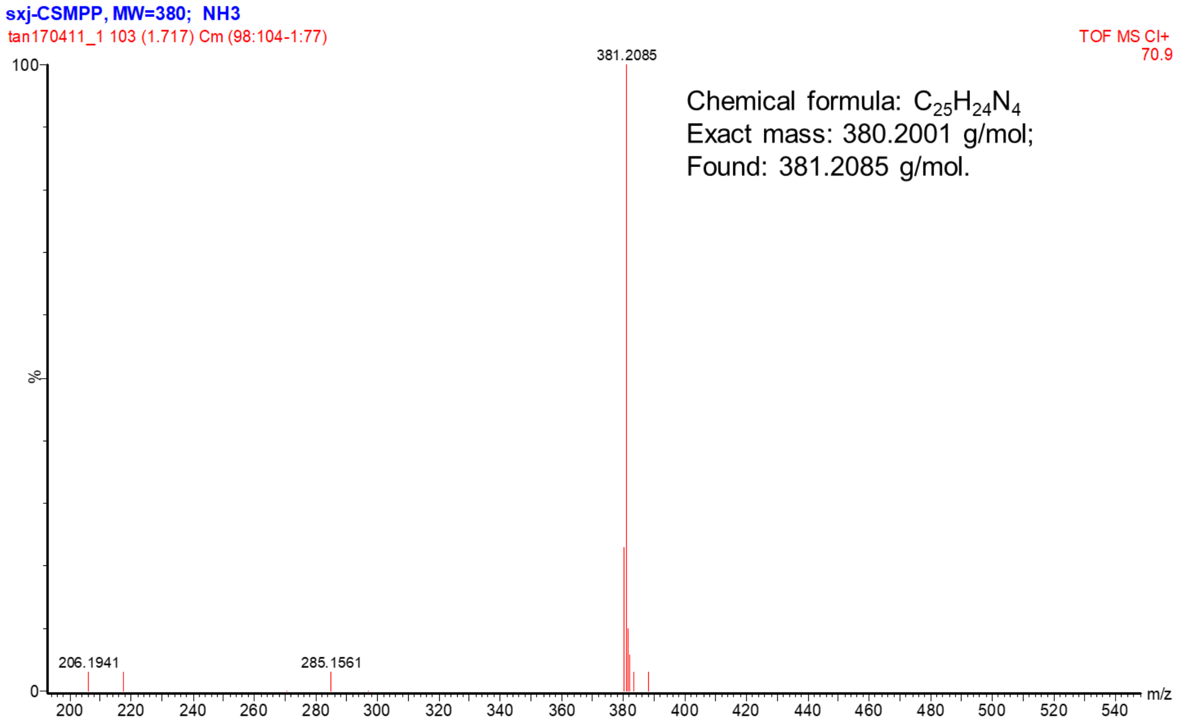

Figure S3. The high-resolution mass spectrum of CSMPP.

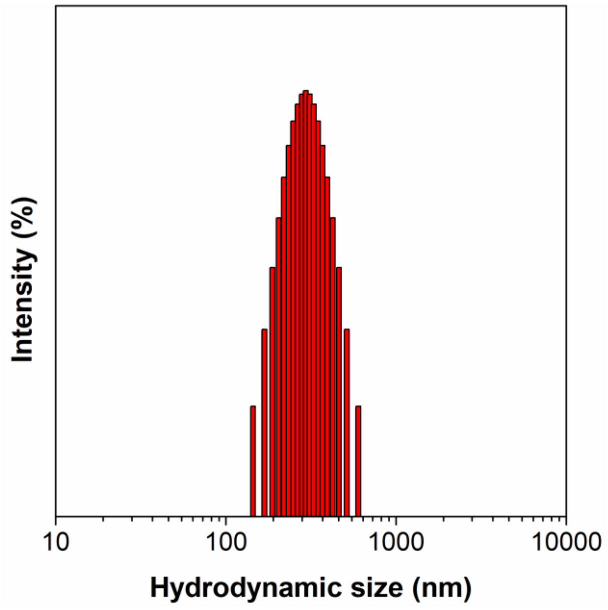

Figure S4. The hydrodynamic diameter of 10 μM CSMPP in acetonitrile/water solution when f_w is 90%. The hydrodynamic diameter of the aggregates is 312±23 nm measured by dynamic light scattering.

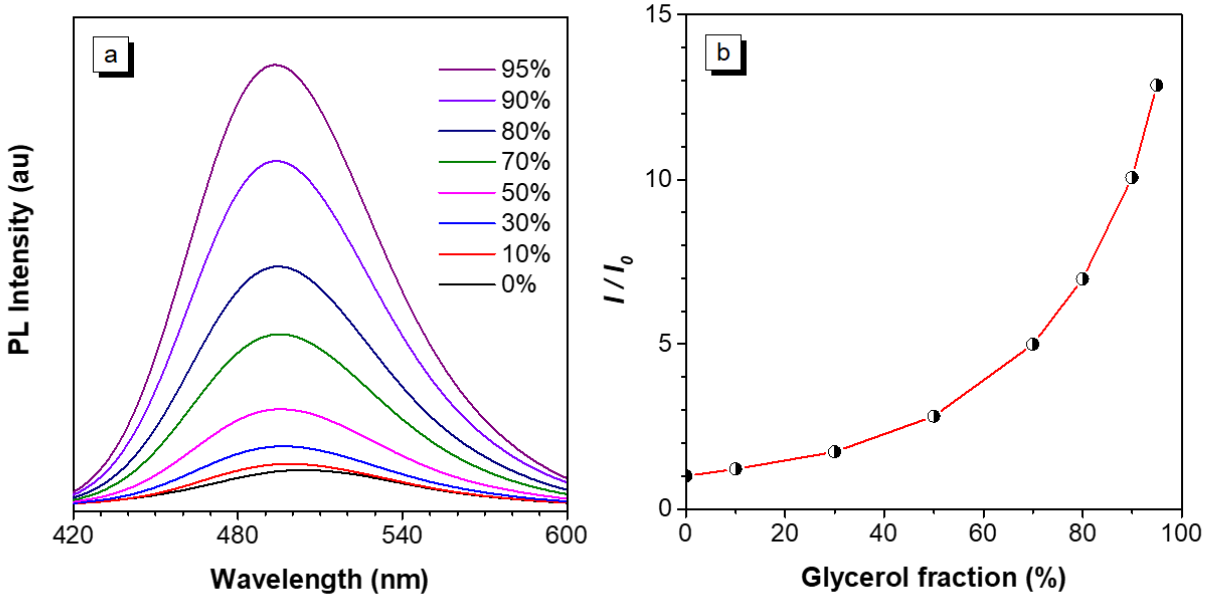

Figure S5. (a) PL spectra of CSMPP in glycerol/ethylene glycol mixtures with different volume fractions of glycerol. (b) Plot of I/I_0 *versus* glycerol fraction. I and I_0 represent the PL intensities at 494 nm in glycerol/ethylene glycol mixture with a specific glycerol fraction and in pure ethylene glycol, respectively. The concentration of CSMPP is 10 μM. Excitation wavelength is 365 nm.

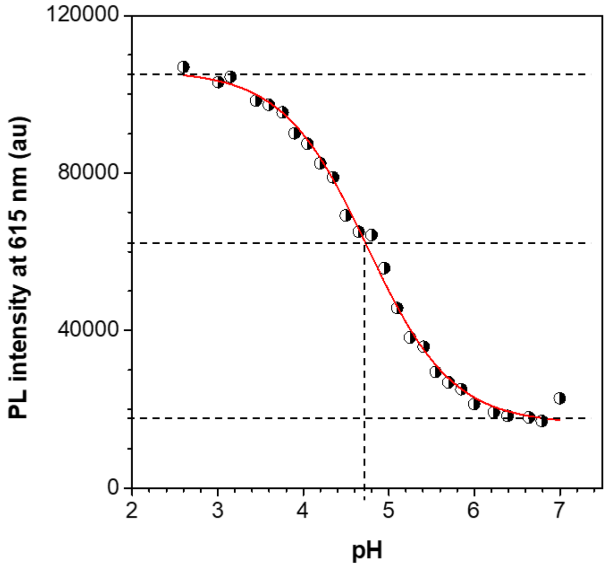

Figure S6. The fluorescence intensity at 615 nm as a function of pH. The corresponding pH when the PL intensity changes by half represents pK_a. The calculated pK_a is 4.75 ± 0.02.

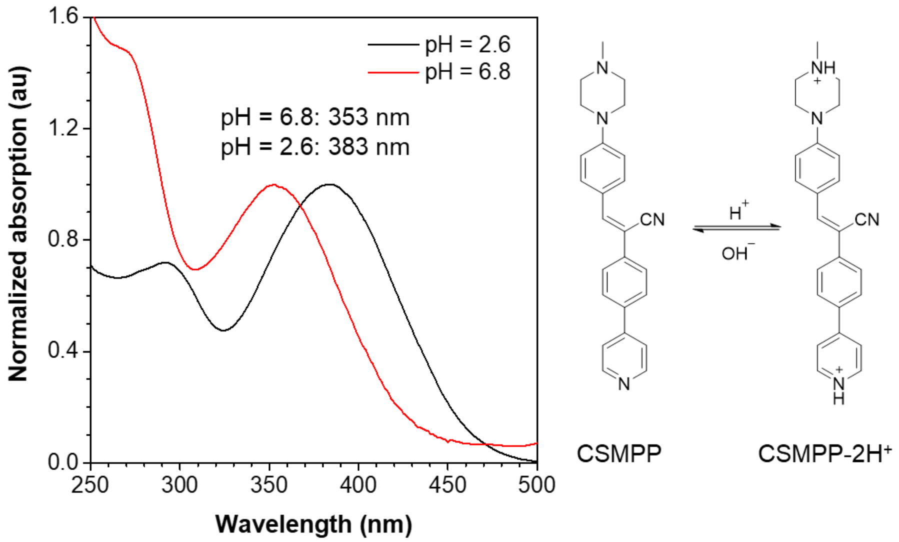

Figure S7. The normalized absorption of CSMPP in buffers at pH 6.8 and pH 2.6. And the corresponding structures of CSMPP in pH 6.8 and pH 2.6.

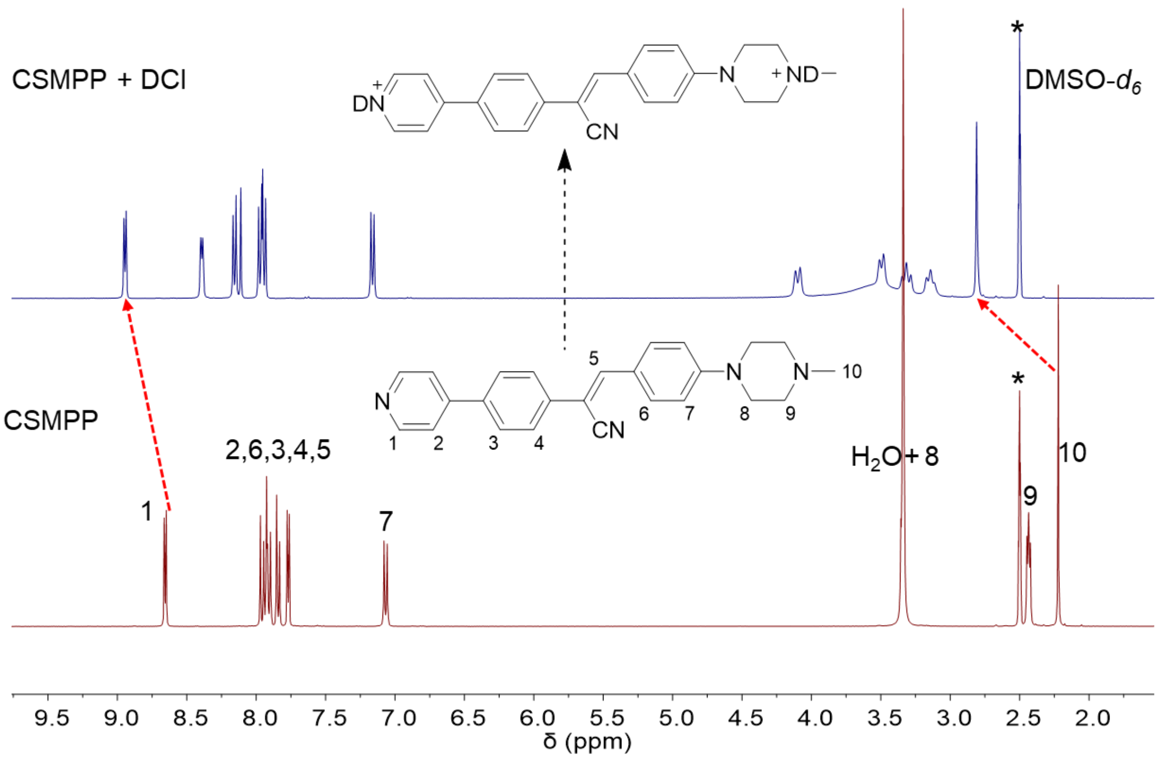

Figure S8. ^{1}H NMR spectra of CSMPP before and after addition of an excess of deuterium chloride (2.5 equiv.) into DMSO-d_6 solution.

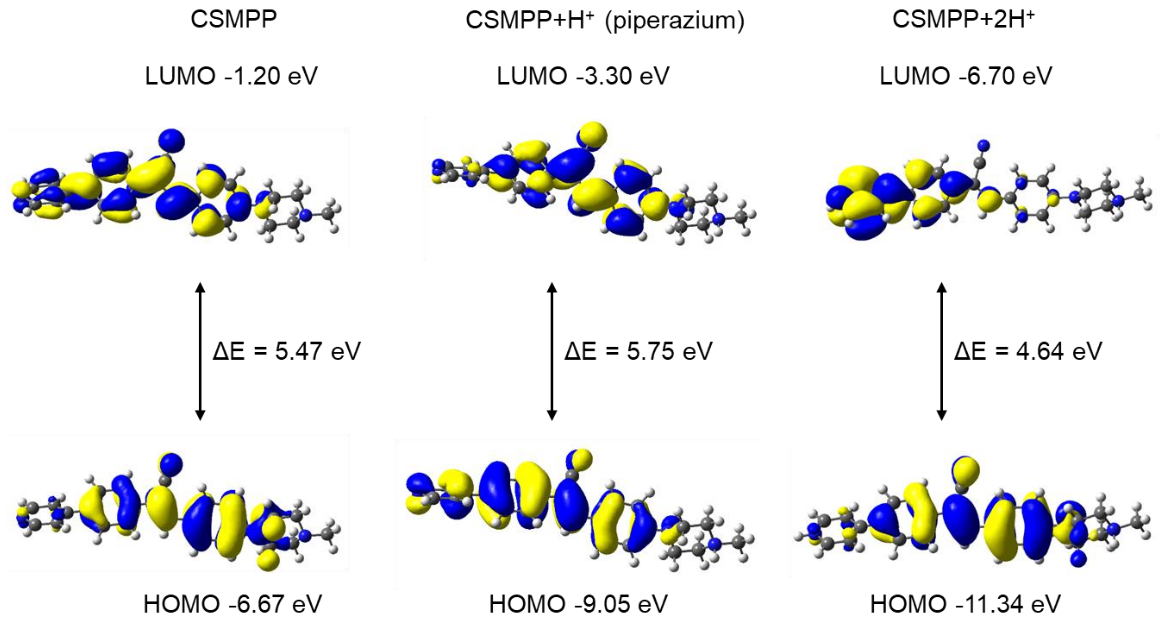

Figure S9. Electron cloud distributions of the HOMO and LUMO of the ground state of CSMPP before and after acidification based on Frontier molecular orbitals optimized by M062X/6-31G(d,p) level.

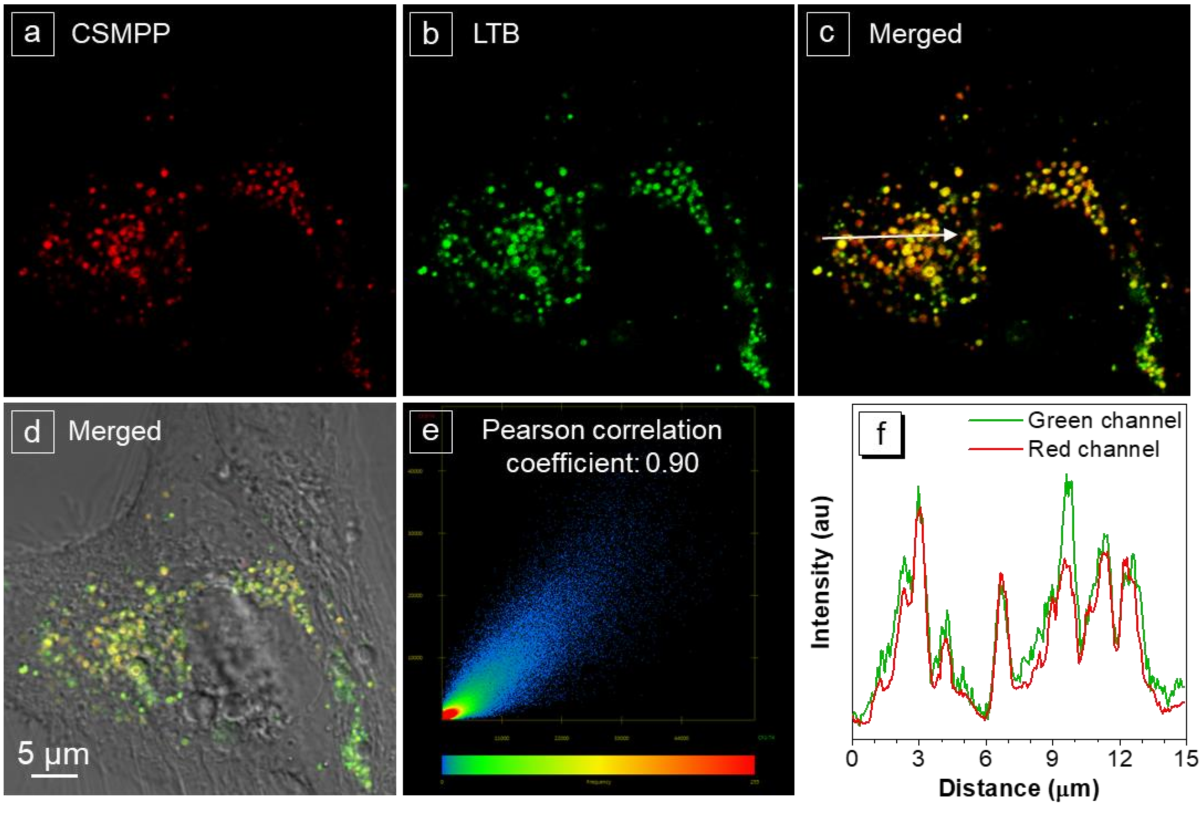

Figure S10. Confocal images of ARPE-19 cells stained with 240 nM LysoTracker Blue (LTB) for 10 min and then co-stained with 1.5 μM CSMPP for 10 min. Fluorescent images of (a) CSMPP;

(b) LTB; (c) merged a and b; (d) merged brightfield with a and b; (e) the signal distribution diagram of the red channel for CSMPP and the green channel for LTB; (f) the fluorescence intensity profile extracted from the white arrow line in image c. Conditions: for CSMPP, λ_{ex} = 405 nm, λ_{em} = 480–700 nm. For LTB, λ_{ex} = 405 nm, λ_{em} = 400–440 nm. Scale bar is 5 μm.

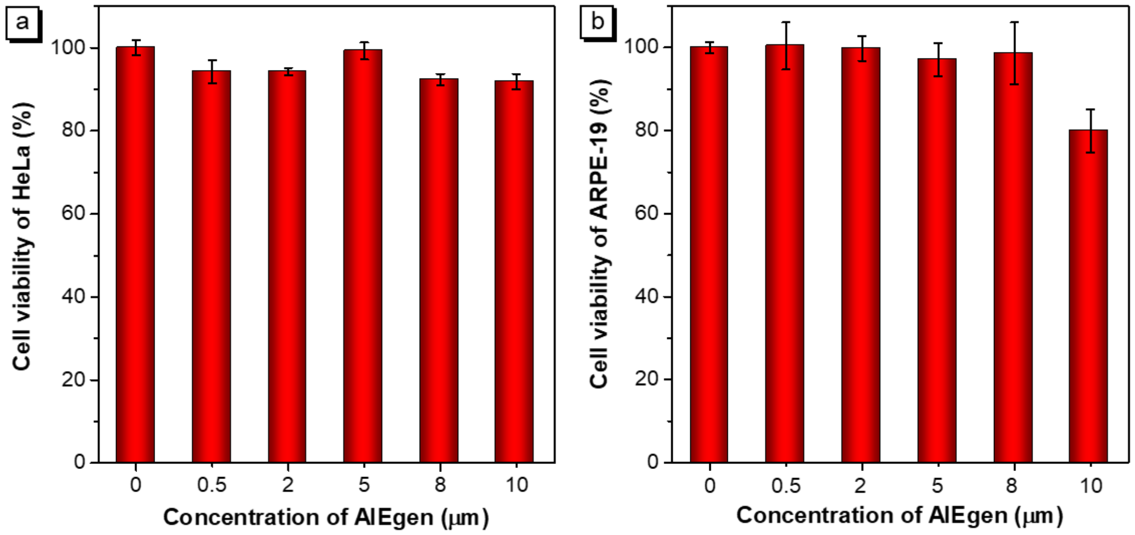

Figure S11. Cell viability of (a) HeLa and (b) ARPE-19 cells incubated in culture medium containing different concentrations of CSMPP for 24 h. Data are the mean ± SD (n = 4).

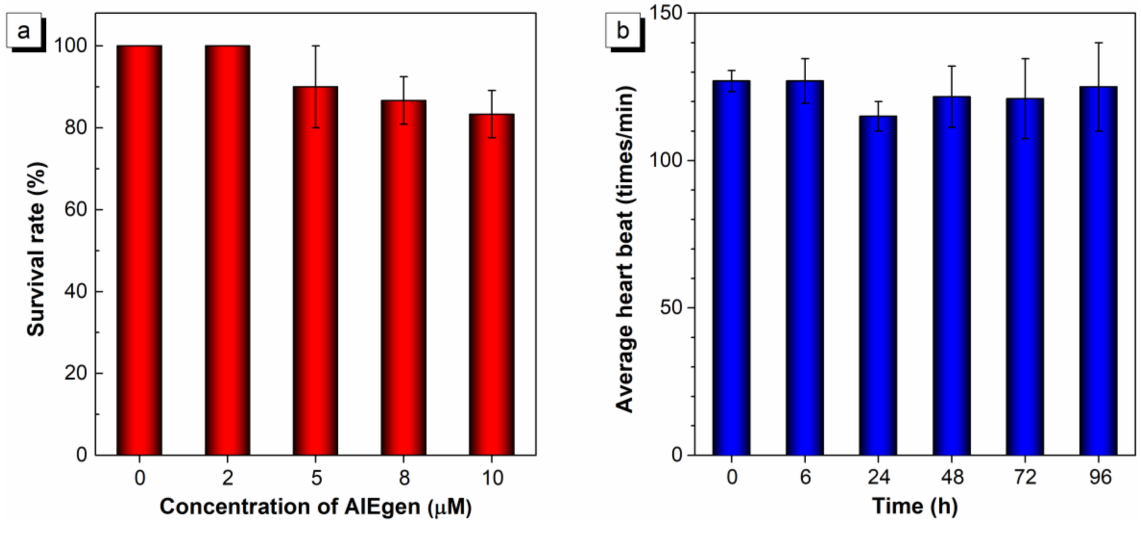

Figure S12. The biocompatibility of CSMPP to medaka larvae was assessed by (a) measuring the survival rate after exposing larvae to different concentrations of CSMPP for 96 h and by (b) recording the mean heartbeat of larvae at different times after feeding them with 5 μM CSMPP for 4 h. Three replicates were included in each concentration treatment, and each replicate contained 10 individual larvae. The heartbeat of fifteen larvae was recorded each time. Data are the mean ± SD.

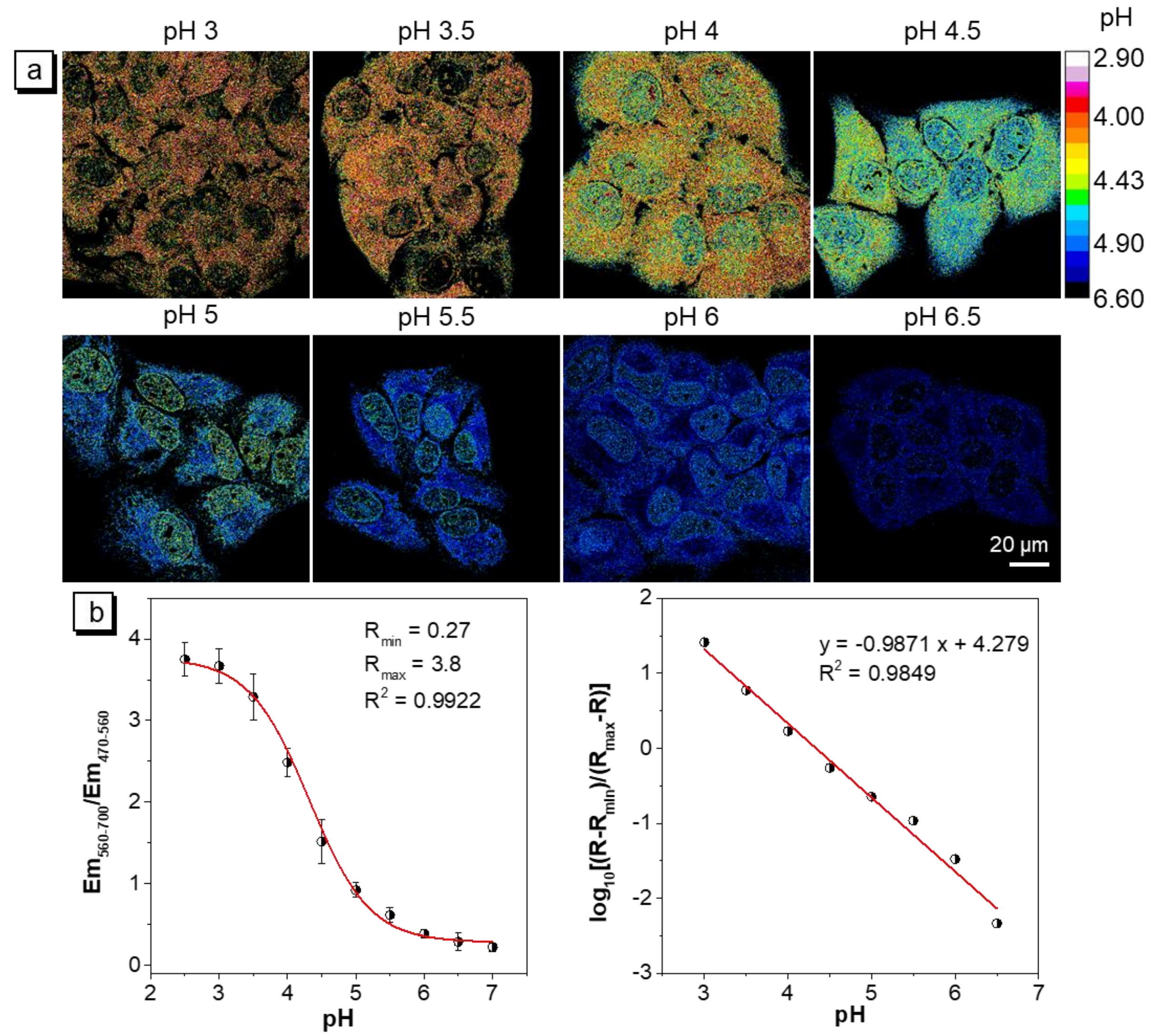

Figure S13. (a) The pH calibration of CSMPP probe in HeLa cells captured by using CLSM with a 63x oil objective lens. Cells were first stained by 3 µM CSMPP for 1 h at 37 ºC. Then, cells were equilibrated in pH calibration buffers containing 12 µM nigericin and 5 µM monensin for 8 min at 25 ºC. For green channel: λ_{em} = 470–560 nm; For red channel: λ_{em} = 560–700 nm. λ_{ex} = 405 nm. The ratiometric image was acquired by dividing the red channel by the green channel with Image J software. Scale bar is 20 µm. (b) The mean ratio of $E_{m\text{-}red}/E_{m\text{-}green}$ as a function of pH. Mean ± SD between three images were presented. The calibration curve was fitted based on the relationship between the mean ratio, R_{min}, R_{max} and the pH, whose calculation equation is presented in equation 2.

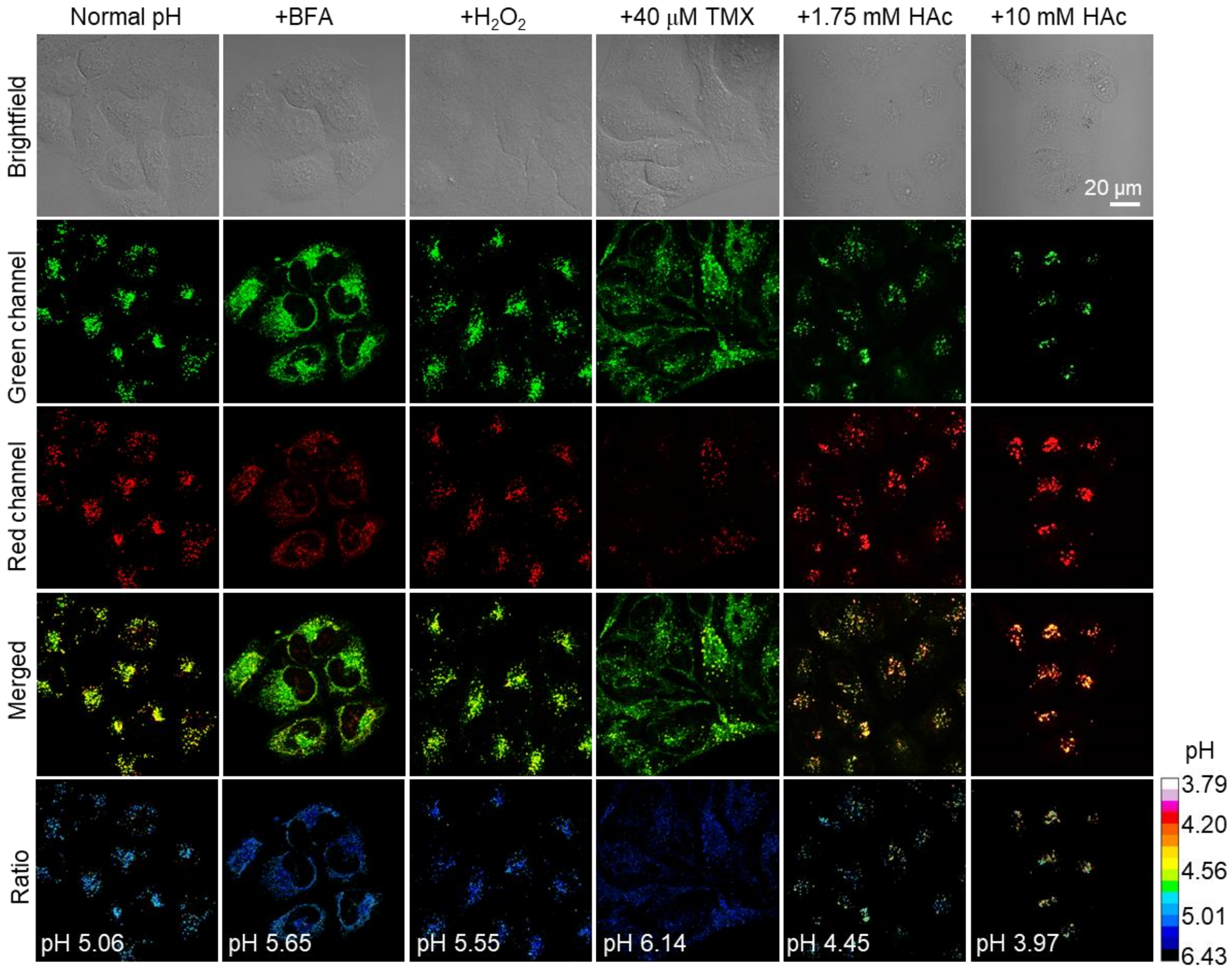

Figure S14. The CSLM images after HeLa cells were stimulated by chemical stimulants, including brightfield, green channels, red channels, merged two-channel images, and ratiometric images showing the lysosomal pH distribution. Chemical stimulants: normal lysosomal pH without stimulants; 50 nM bafilomycin A1 (BFA) incubated for 30 min, 0.1 mM H_2O_2 incubated for 30 min; 40 μM tamoxifen (TMX) incubated for 15 min; 1.75 mM and 10 mM acetic acid (HAc) incubated for 8 min at 37 ^{o}C. The captured confocal fluorescence images of the red and the green channels were analyzed by using Image J software to acquire the ratiometric images. Excitation: 405 nm. The emission of the green channel at 470−560 nm and the red channel at 560−700 nm were collected. Scale bar is 20 μm.

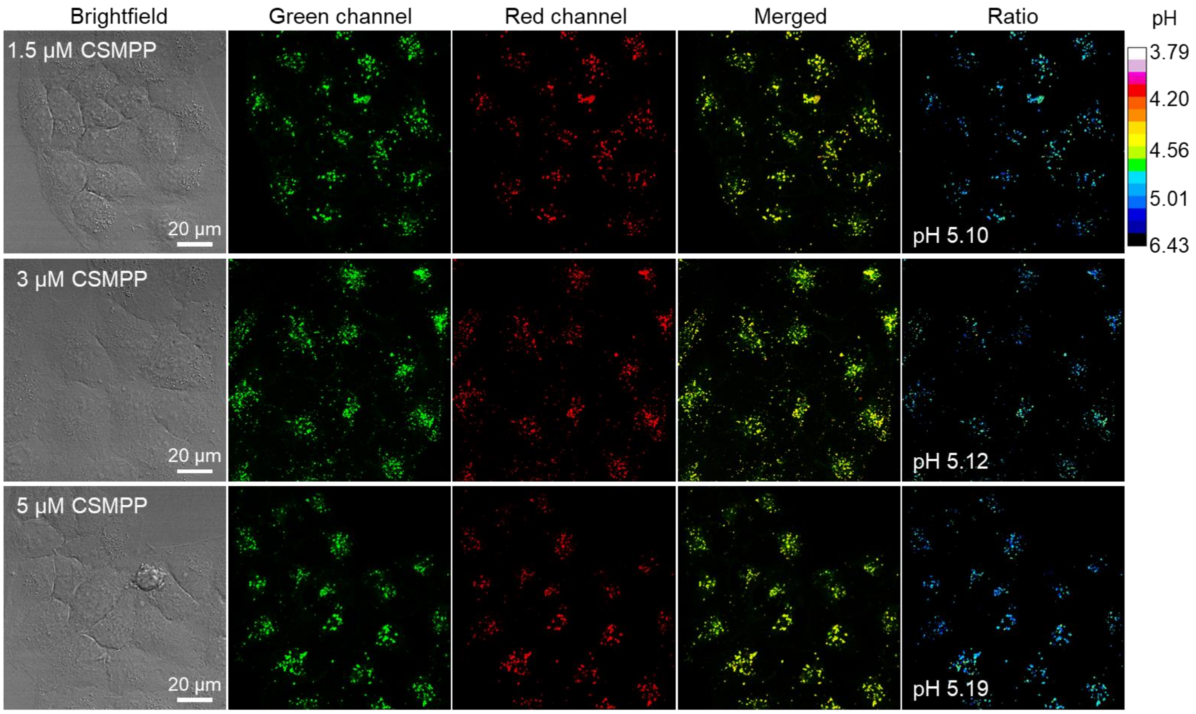

Figure S15. The lysosomal pH of HeLa cells after being incubated by different concentrations of CSMPP for 5 min. Excitation wavelength is 405 nm. The emission of the green channel at 470−560 nm and the red channel at 560−700 nm were collected. The ratio image was acquired by dividing the red channel with the green channel using Image J software. Scale bar is 20 μm.

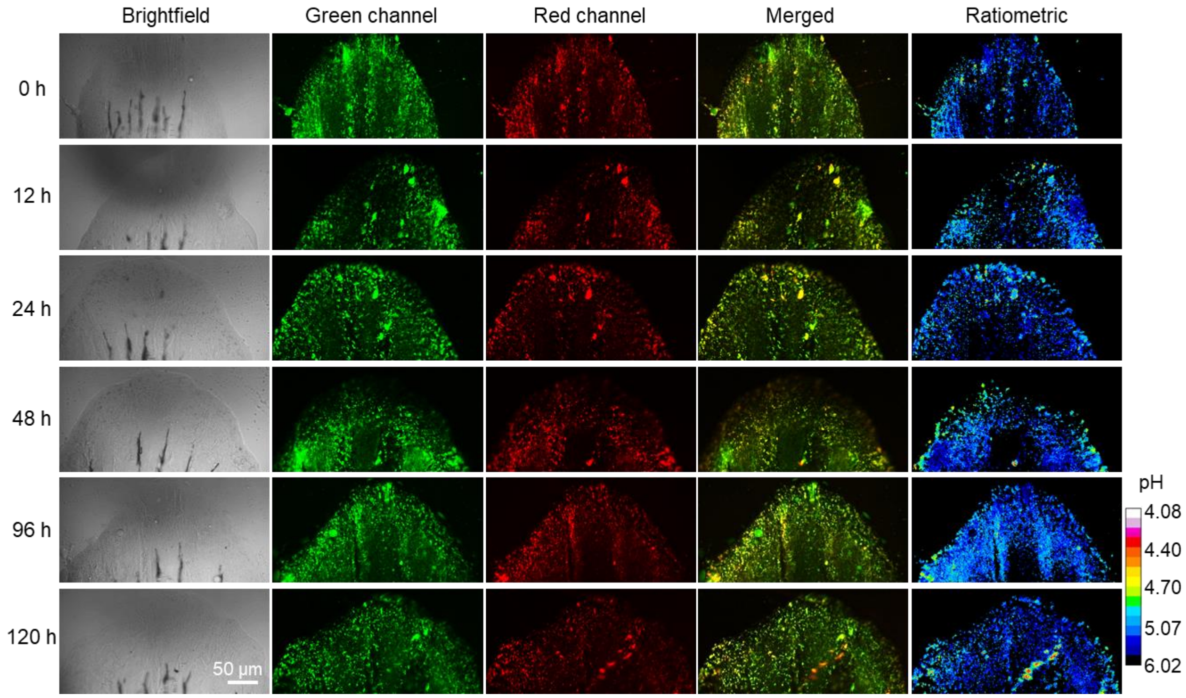

Figure S16. The CSLM images of medaka larva's caudal fin without amputation at different times after being fed with 5 μM CSMPP for 4 h, including brightfield, green channels, red channels,

merged two-channel images, and ratiometric images showing the lysosomal pH distribution. For the green channel: λ_{em} = 416–555 nm; for the red channel: λ_{em} = 557–704 nm. λ_{ex} = 405 nm. The ratiometric images of $E_{m\text{-red}}/E_{m\text{-green}}$ were analyzed by using Image J software based on the fluorescence images. As a comparison with the amputated medaka larvae, the medaka larvae without amputation after being fed with CSMPP were photographed at the same time as that of photographing the amputated ones. The hours shown in the figure were named based on the hours after amputation. And "0 h" means the time after being fed with CSMPP and before amputation. Scale bar is 50 μm.

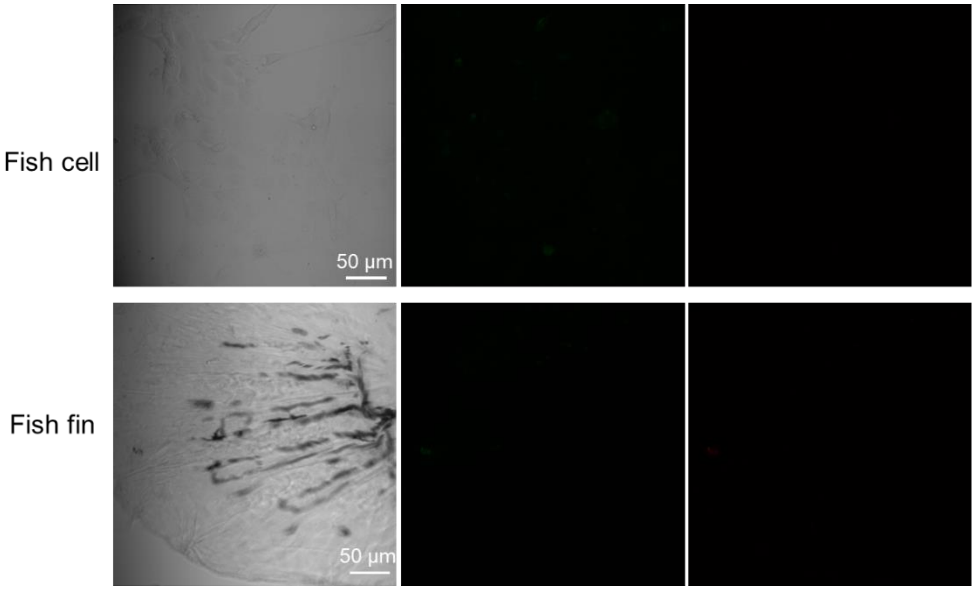

Figure S17. The confocal images of fish cells and the caudal fin without being incubated by dyes. The green channel (416−555 nm) and the red channel (557−704 nm) images were captured with the same imaging condition as when tracking lysosomal pH. Scale bar is 50 μm.

Table S1. Crystal data and structure refinement for CSMPP.

Bond precision:	C-C = 0.0020 A		Wavelength=1.54184
Cell:	a=6.6601(4)	b=7.4588(4)	c=23.5097(15)
	alpha=94.931(5)	beta=90.774(5)	gamma=105.499(5)
Temperature:	100 K		
	Calculated		Reported
Volume	1120.43(12)		1120.42(12)
Space group	P -1		P -1
Hall group	-P 1		-P 1
Moiety formula	C25H24N4, 3(H2O)		C25H24N4, 3(H2O)
Sum formula	C25H30N4O3		C25H30N4O3
Mr	434.53		434.53
Dx,g cm^{-3}	1.288		1.288
Z	2		2
Mu (mm-1)	0.691		0.691
F000	464.0		464.0
F000'	465.36		
h,k,l_{max}	7,8,28		7,8,28
Nref	4026		3945
T_{min},T_{max}	0.920,0.933		0.884,1.000
$T_{min'}$	0.920		

Correction method= # Reported T Limits: T_{min}=0.884 T_{max}=1.000 AbsCorr = MULTI-SCAN

Data completeness= 0.980	Theta(max)= 67.473
R(reflections)= 0.0394(3173)	wR2(reflections)= 0.0981(3945)
S = 1.008	Npar= 299
CCDC	1938403

3 References

1. G. Grynkiewicz, M. Poenie, R. Y. Tsien, *J. Biol. Chem.*, 1985, **260**, 3440-3450.

4 Author Contributions

Dr. Xiujuan Shi designed and done most of the experiments, analyzed the data and wrote the manuscript. Mr. Neng Yan raised the fish, done all the experiments involved of medaka larvae and discussed the data. Dr. Xiujuan Shi and Mr. Neng Yan contributed equally. Dr. Guangle Niu, Dr. Ryan T. K. Kwok and Dr. Jacky W. Y. Lam helped to revise the manuscript. Mr. Simon H. P. Sung helped to synthesize some raw materials. Dr. Guangle Niu and Dr. Zhiyang Liu discussed the data and made suggestions for writing the manuscript. Mr. Junkai Liu did the molecular simulation. Prof. Wen-Xiong Wang supported the medaka larvae and the funding. Dr. Herman H.-Y. Sung and Prof. Ian D. Williams helped to measure and analyze the single crystal structure. Dr. Ryan T. K. Kwok supported the funding. Prof. Benzhong Tang supported the funding, discussed and improved the project, and gave suggestions for writing the manuscript.
